# The influence of emotions on cognitive control: feelings and beliefs—where do they meet?

**DOI:** 10.3389/fnhum.2013.00508

**Published:** 2013-09-19

**Authors:** Katia M. Harlé, Pradeep Shenoy, Martin P. Paulus

**Affiliations:** ^1^Department of Psychiatry, University of CaliforniaSan Diego, La Jolla, CA, USA; ^2^Department of Cognitive Science, University of CaliforniaSan Diego, La Jolla, CA, USA; ^3^Psychiatry Service, VA San Diego Healthcare SystemLa Jolla, CA, USA

**Keywords:** emotion, inhibitory control, Bayesian modeling, ideal observer model

## Abstract

The influence of emotion on higher-order cognitive functions, such as attention allocation, planning, and decision-making, is a growing area of research with important clinical applications. In this review, we provide a computational framework to conceptualize emotional influences on inhibitory control, an important building block of executive functioning. We first summarize current neuro-cognitive models of inhibitory control and show how Bayesian ideal observer models can help reframe inhibitory control as a dynamic decision-making process. Finally, we propose a Bayesian framework to study emotional influences on inhibitory control, providing several hypotheses that may be useful to conceptualize inhibitory control biases in mental illness such as depression and anxiety. To do so, we consider the neurocognitive literature pertaining to how affective states can bias inhibitory control, with particular attention to how valence and arousal may independently impact inhibitory control by biasing probabilistic representations of information (i.e., beliefs) and valuation processes (e.g., speed-error tradeoffs).

## Introduction

How do feeling and thinking influence one another? From our subjective experience, and systematic behavioral research, we know that affective states profoundly influence cognitive functions, in both facilitative and antagonistic manners depending on the context. This relationship between affect and behavior is not surprising, given the extensive interactions between the physiological and interoceptive manifestation of emotion (Craig, 2002; Paulus and Stein, 2006) and cognitive control networks (Botvinick et al., 2001; Pessoa, 2009). In particular, impairments in critical executive faculties such as inhibitory control (Miyake et al., 2000) are tightly linked to clinical disorders involving pervasive emotional states and difficulty in regulating emotion. However, little is known about the specific computational and cognitive processes underlying such interactions between emotion and inhibition. Thus, understanding precisely how emotion is integrated into core executive functions, such as inhibitory control, is essential not only for cognitive neuroscience, but also for refining neurocognitive models of psychopathology.

In this review, we propose a computational framework to conceptualize emotional influences on cognition, focusing in particular on inhibitory control. We build upon research suggesting that a wide range of apparently distinct cognitive faculties can be unified under a common “ideal observer” framework of decision-making and dynamic choice. Rational observer models have been applied widely to the study of choice in uncertain environments, and to identify potential neural markers of the iterative processes of belief update underlying such models (Hampton et al., 2006; Behrens et al., 2007). Subsequent modeling work showed that such a framework is readily adapted to various aspects of executive function, including attentional and inhibitory control (Yu and Dayan, 2005; Yu et al., 2009; Shenoy and Yu, 2011; Ide et al., 2013). In particular, this literature suggests that apparently distinct faculties in inhibitory control can be folded into a single framework where subtle differences in task contexts are reflected in their influence on components of the framework, giving rise to the diversity of observed behavior. Building on this research, we argue for an *emotion-aware* rational observer model of inhibitory control, where emotions serve as additional *context* for the computations underlying behavior. Indeed, previous research has explored the idea of emotion providing information about one's internal state to the executive system. Therefore, emotion can be considered part of the information that along with external stimuli is integrated to perform controlled actions (Schwarz and Clore, 1983; Forgas, 2002). Such biases appear to be mediated by mood-congruent effects on memory [i.e., priming access to and retrieval of mood-congruent concepts and outcomes (Bower, 1981)] and interoceptive processes [i.e., conveying information about ones' valuation of / disposition toward choice options (Schwarz and Clore, 1983)]. Therefore, here we propose a wider role for emotional context in cognition, and consider how it may affect beliefs and action valuation in much the same way as other environmental constraints and information do. We consider such interactions within the confines of our decision-making framework for inhibitory control, thereby allowing us to relating emotion directly to other, well-understood computational principles underlying cognition.

In the following sections, we first review Bayesian ideal observer models of inhibitory control using a shared computational framework to guide discussion. The following section is organized into two parts, distinguishing two broad types of computational elements that may be modulated by emotion, namely a) probabilistic computations (i.e., reflecting individuals' beliefs about the frequency of certain events or actions) and b) valuation computations (i.e., reflecting the value or cost associated with potential outcomes and actions). To maximize the theoretical usefulness of our model, we further opt for a dimensional decomposition of emotion rather than considering the impact of multiple separate emotions on inhibitory control. Thus, within this computational framework, we distinguish two empirically validated dimensions of emotion with distinct physiological markers (Lang et al., 1997; Tellegen et al., 1999; Davidson, 2003): valence or motivational tendency (i.e., positive/appetitive vs. negative/aversive tone), and arousal (or emotional salience or intensity). We acknowledge that while valence and motivational tendency are theoretically different constructs and their respective validity still a matter of debate, they have a high degree of overlap in most emotional states. Specifically, most negative emotions are withdrawal based and positive emotions are approach based, with one notable exception being anger (Harmon-Jones and Allen, 1998). Given the limited number of studies specifically attempting to dissociate the effects of these dimensions on inhibitory control, it was not feasible to distinguish between them in the present review. However, we address this distinction in our proposed framework by considering two mediating computational mechanisms through which valence and arousal may infuse the computational underpinnings of inhibitory control, namely outcome vs. action related computational processes. In support of this distinction, separate neural markers have been linked to anticipation of an outcome vs. the appetitive or aversive disposition or *drive* toward a particular outcome [i.e., action tendency; (Breiter et al., 2001; Miller and Tomarken, 2001; Knutson and Peterson, 2005; Boksem et al., 2008)]. Thus, from a computational and neural perspective, these outcome and action tendencies may emerge from very different underlying components. Therefore, we evaluate valence and arousal with respect to their potential impact on (a) action and outcome expectancies (i.e., probabilistic predictions), as well as (b) action and outcome valuation (i.e., relative importance of these events in the decision policy).

We propose several hypotheses linking these affective dimensions (and their attendant behavioral influences) to specific components of the computational framework. Based on the AIM model of affect infusion and extensive literature pointing to a strong interdependence between hedonic valence and the behavioral activation/inhibition system (Niv et al., 2007; Huys et al., 2011; Guitart-Masip et al., 2012), we conjecture that the valence dimension may promote both valence-congruent effects on outcome-related computations and motivational effects on activation and inhibition. In contrast, arousal may primarily modulate action cancellation expectancies and, at higher thresholds, have a more indirect impact on computational processes by redirecting attentional resources and impairing prefrontal cortical function (Arnsten, 2009a). These hypotheses suggest testable, quantitative relationships between emotional state and inhibitory control.

## Models of inhibitory control

### Cognitive models of inhibitory control

Much of the theoretical literature on inhibitory control focuses on the contrast between action and inhibition and different aspects of inhibition such as attentional and behavioral inhibition. Accordingly, the literature suggests separate functional instantiation of these putative processes, both in abstract cognitive models and in proposals for neural architectures. For instance, several articles propose a *conflict* model of inhibitory control, where certain stimuli may activate multiple action plans, thus generating conflict between competing responses (Botvinick et al., 2001). This notion of conflict has been explored at the neural level using a contrast between trial types in a variety of tasks such as the Stroop task (Barch et al., 2000; Macleod and Macdonald, 2000), the flanker task (Botvinick et al., 1999) the Simon task (Peterson et al., 2002; Kerns, 2006), and the Stop Signal task (Brown and Braver, 2005). As an example, in the Eriksen task, incongruent stimuli are thought to generate conflict between the responses associated with central and flanker stimuli, resulting in behavioral differences and corresponding neural activation. Other work has drawn on the empirical data to suggest architectures for *monitoring and resolution* of conflict (Botvinick et al., 2001; Botvinick, 2007) and error (Brown and Braver, 2005), where specific areas of the brain monitor any resulting conflicts or errors in order to adjust behavior appropriately. Closely related work considers models of the specific *underlying processes* that may give rise to action and inhibition, respectively. For instance, in the stop signal task, the influential *race model* of stopping (Logan and Cowan, 1984) suggests that behavior is an outcome of a race between finishing times of “stop” and “go” processes, corresponding to inhibition and response, respectively. A rich literature has explored potential instantiations of this race model at various levels of neural activity: from neural firing rates (Hanes et al., 1998; Paré and Hanes, 2003; Stuphorn et al., 2010) to population activity in specific brain regions such as the IFC (Aron et al., 2004) to putative “stopping circuitry” involved in inhibition of action (Aron et al., 2007a).

The consensus in much of this work is of a contrast between *inhibition* and *action*, with potentially different mechanisms and neural circuitry involved in these functions. Further, individuals are thought to exercise different kinds of inhibition, depending on the task demands. From this perspective, behavioral and neural measures of performance in inhibitory control tasks measure the *relative efficacy* or dysfunction of these competing systems, and each such measure may reflect the performance of a different subsystem. For instance, (Eagle et al., 2008) compare and contrast the go/nogo and stop signal tasks from behavioral, neural and pharmacological perspectives, suggesting a dissociation between different *kinds* of behavioral inhibition: “restraint” (the go/nogo task) and “cancellation” (the stop signal task). Other work (Nee et al., 2007; Swick et al., 2011) explores, from a neural perspective, the possibility of shared circuitry in various inhibitory control tasks.

In contrast, recent work explores the possibility of studying inhibition using rational observer models, where all behavioral outcomes (various responses, or the absence of a response) are produced by a single, rational (i.e., reward-maximizing) decision-making framework. In the rest of this section, we outline the proposed framework using different inhibitory control tasks as examples. The framework promises to unify the wide variety of behavioral and neural results from studies of different inhibitory control tasks, currently ascribed to different functional systems. In addition, this unifying perspective may suggest how other, apparently distinct, influences such as emotion, may also be integrated into a computational decision-making perspective.

### Inhibitory control as rational decision-making

**Figure 1:**
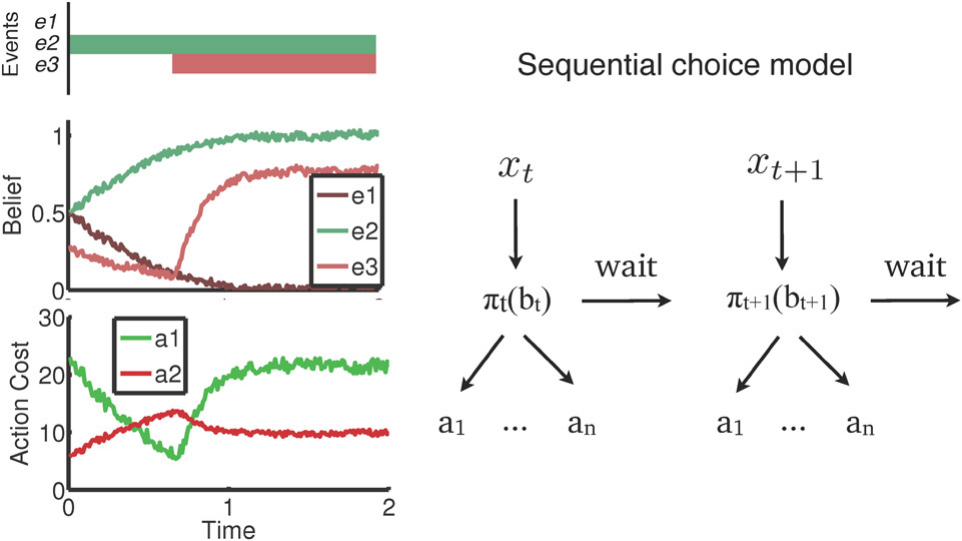
**Rational decision-making in inhibitory control.** The figure abstracts out ideas common across recent decision-making models for inhibitory control into a single framework. **Left**: an example where task-relevant events e1 and e2 are mutually exclusive (e.g., a forced choice stimulus), and e3 occurs at some later point in time. Sensory evidence from these events are gradually reconciled with prior expectations to form a noisy, evolving belief, or subjective probability, about whether the event occurred. These beliefs form the basis of an ongoing valuation of, and selection between, available actions. **Right**: A representation of this sequential decision-making process. At each time point, noisy sensory inputs (*x*_i_) are incorporated into beliefs (*b*_i_), which are transformed into a choice between actions (a1,… an, wait) based on the decision policy (∏).

A recent body of work (Yu et al., 2009; Shenoy and Yu, 2011, 2012; Shenoy et al., 2012) recast behavior in a wide variety of inhibitory control tasks as *rational* (i.e., reward-maximizing) tradeoffs between uncertainty and the cost of available actions. This cost-benefit tradeoff is an *ongoing* decision-making process that unfolds over time as noisy sensory inputs are processed, and reconciled with prior expectations about possible outcomes. A general outline of the decision-making framework is shown in Figure 1. The figure shows an example where certain events in the real world that are task-relevant (e1,…, e3, top panel) are processed gradually over time and represented as *beliefs* or probabilities (middle panel). In the example, e1 and e2 are mutually exclusive events (for instance, a forced-choice stimulus), whereas e3 may or may not occur at some subsequent time. Note that this simple representation captures the general dynamics of most of the discussed inhibitory control tasks. The beliefs (*b*_*t*_) shown in the figure represent the evolving degree of uncertainty an individual has about the state of the world—e.g., has e3 occurred already? Such beliefs are, naturally, influenced by prior expectations. For example, the initial anticipation that e3 might occur is tempered by the initial lack of sensory evidence, whereas subsequent occurrence of the event is quickly reflected in the belief. Based on the belief state, subjects have to weigh the costs associated with various available actions, and select repeatedly between them. Note that in the model, *inaction* is also an available “action,” with an attendant cost determined by the environment, and an advantage of acquiring more information for decision-making. The entire decision-making schematic is depicted in the right panel of Figure 1.

Below, we illustrate how the framework may be applied to a variety of inhibitory control paradigms. Through this exercise, we aim to demonstrate that (1) different inhibitory control tasks may be understood and interpreted using the same shared framework, and (2) the apparent idiosyncrasies of behavior in the tasks reflect subtle differences in the task contexts, and draw focus on specific components of the proposed model. The first two sections address belief formation and updating, which we show can occur within trial (i.e., based on increased certainty about relevant sensory information) but also on a trial-to-trial basis (i.e., based on cumulative experience with the task). The third section introduces valuation processes as a framework for understanding speed accuracy tradeoffs.

#### Sensory disambiguation: conflict and resolution

**Figure 2:**
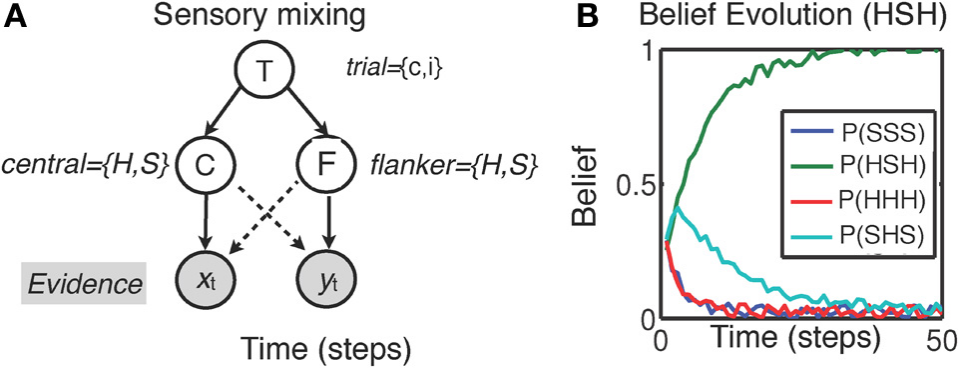
**Sensory disambiguation in the Eriksen task (Yu et al., 2009). (A)** The model assumes that sensory inputs *x*_*t*_ (central stimulus) *y*_*t*_ (flanker) are mixed. Responding to the central stimulus C necessitates processing all sensory information and simultaneously decoding both the central stimulus and trial type T (*T* = *c* on congruent trials; *T* = *i* on incongruent trials) which depends on disambiguation of central and flanker (F) stimuli; H,S = stimulus type. **(B)** The corresponding Bayesian inference process (schematic) quickly discovers that the trial has an incongruent stimulus, but decoding the central stimulus identity may take longer due to featural mixing and potentially higher prior expectations of encountering congruent trials (i.e., β > 0.5).

We illustrate the influence of sensory processing models on decision making and inhibitory control using the example of interference paradigms introduced above. These tasks all share a critical similarity in that each one sets up a mismatch between two different features of a perceptual stimulus—i.e., information contained in the features may be *congruent* or *incongruent* with each other. The tasks, however, require a response based only on a single stimulus feature. In each of the tasks, subjects are more error-prone and slower to respond on incongruent trials. This difference has been attributed to various aspects of cognitive processing such as *attentional* or *cognitive* inhibition in terms of suppressing irrelevant information (Stroop & Eriksen tasks), or response conflict (Simon task). Instead, behavior in each of these tasks can be reinterpreted as a process of *within-trial* sensory disambiguation and belief update. In particular, (Yu et al., 2009) proposed that human sensory processing may have a “compatibility bias,” where visual features are assumed to vary smoothly over space. This bias could potentially be acquired through experiential or evolutionary means. For instance, in the Eriksen task, this assumption may manifest itself via mixing of sensory evidence between central (C) and flanker (F) stimuli, as illustrated in Figure 2A (adapted and simplified from Yu et al., 2009). The figure suggests that, although the relevant sensory evidence (*x*_*t*_) should only depend on the central stimulus (solid line), perceptual processing is nevertheless affected by flanker stimuli (*y*_*t*_). As a consequence, decoding the central stimulus identity necessitates also decoding the trial type T (congruent or incongruent). Thus, in the proposed framework, the sensory processing that unfolds over time is tasked with disambiguating the trial type and stimulus identity in a *joint* belief state as follows:

(1) $$P\{C,T|X_{t},Y_{t}\}\propto p(x_{t}|C)p(y_{t}|C,T)P\{C,T|X_{t\;-\;1},Y_{t\;-\;1}\}$$

Here, the central stimulus identity (*C* = “*H*” or “*S*”) and the trial type T (*T* = *c* for congruent or *T* = *i* for incongruent) are both discrete and binary valued. The joint distribution in Equation 1 incorporates all the information gathered from previous observations (*x*_*t*_, *y*_*t*_). This iterative process is initialized by a prior distribution representing prior beliefs about the prevalence of congruent trials [β = *P*(*T* = *c*|*X*_0_, *Y*_0_)] and the possible central/flankers stimuli configurations (e.g., “SSS” vs. “HHH” for congruent trials, and “SHS” vs. “HSH” for incongruent trials, based on a simplified case of only 2 flankers, see Figure 2B). To make a perceptual decision about the central stimulus *C*, the total (marginal) probability *P*(*C* = *H*|*X*_*t*_, *Y*_*t*_) is computed by summing the joint probabilities over the uncertainty about congruency (i.e., *T* = *c* and *T* = *i*):

(2a) $$P\left(C=H|X_{t},Y_{t}\right)=P\left(C=H,T=c|X_{t},Y_{t}\right)+P\left(C=H,T=i|X_{t},Y_{t}\right)$$

Since the stimulus identity only assumes two values (“*H*” or “*S*”), the probability of C being S is simply:

(2b) $$P\left(C=S|X_{t},Y_{t}\right)=1-P\left(C=H|X_{t},Y_{t}\right)$$

It can be shown that the *optimal* decision policy compares these two marginal probabilities against a decision threshold *q*, and decides that the target is *H* if *P*(*C* = *H*|*X*_*t*_, *Y*_*t*_) > *q*, or *S* if *P*(*C* = *S*|*X*_*t*_, *Y*_*t*_) > *q*. If these conditions are not met, the policy continues observing the input data. On congruent trials, the reinforcing effect of the irrelevant flanker features lead to fast, more accurate responses, whereas incongruent trials require much longer to decode due to the corrupting influence of the flankers on stimulus disambiguation. So, for instance, the “compatibility bias” shown by subjects may manifest itself through a skewed *prior belief* in the probability of compatibility (i.e., β > 0.5; see Figure 2B). As outlined in part III, we propose that emotional states may influence sensory processing (hence behavioral performance) via such altered prior probability distributions.

#### Belief updating: learning to anticipate

In addition to the *within trial* evolution of beliefs observed during sensory disambiguation, recent work (Ide et al., 2013) suggests that prior expectations and belief updating occurring *across trials* also profoundly influence inhibitory control. For example, in a stop signal task, they showed that the immediate experienced history of trial types induced an ever-changing expectation of a stop signal on the upcoming trial, *P*(stop), and that the prior probability successfully predicted subsequent response times and accuracy on the trials. Formally, if *r*_*k*_ is the stop signal frequency on trial k and *s*_*k*_ is the actual trial type (1 on stop trials and 0 on go trials), *P*(stop) is the mean of the predictive distribution *p*(*r*_*k*_|*S*_*k* − 1_), which is a mixture of the previous posterior distribution *p*(*r*_*k* − 1_|*S*_*k* − 1_), and a fixed prior distribution [*p*_0_(*r*)], with α and 1 − α acting as the mixing coefficients, respectively:

(3a) $$p(r_{k}|{\text{S}}_{k-1})=\alpha p(r_{k-1}|{\text{S}}_{k-1})+(1-\alpha )p_{0}(r_{k})$$

where *S*_*k*_ = {*s*_1_,…, *s*_*k*_} with the posterior distribution being updated according to Bayes' Rule:

(3b) $$p(r_{k}|{\text{S}}_{k})\propto P(s_{k-1}|r_{k})p(r_{k}|{\text{S}}_{k-1})$$

Note that the probabilities in Equations 3a,b, as those in Equations 1 [β = *P*(*C, T*|*X*_0_, *Y*_0_)], represent expectancies about the likelihood of encountering various trial types associated with specific action requirements (e.g., frequency of stop trials, congruent trials, etc.), before the onset of each trial. Equations 3a,b show that these expectancies may evolve across trials to form an *iterative prior probability* for the associated action. As we discuss subsequently, while such *action expectancies* are key computational mediators of inhibitory performance, expectations of reward or punishment (i.e., *outcome expectancies*) may be equally relevant to our framework as they tend to co-vary with emotional sates. For instance, the use of inherently rewarding or punishing stimuli as trial type cues (i.e., paired with go or stop action requirement) may provide additional context to bias estimations of trial type probabilities (e.g., which could be modeled by an additional fixed prior that influences stimulus expectation).

#### Speed-accuracy tradeoffs: go bias and rational impatience

Focusing on inhibition and action valuation, we now introduce a general cost function framework for perceptual decision-making tasks as an example of how action valuation impacts measures of inhibition. Subsequently, we focus on two variants of this perceptual decision-making framework, namely the 2-alternative forced choice (2AFC) task (e.g., flanker) and the go/no-go task. As indicated in Figure 1, the moment-by-moment belief state generated through sensory processing results in estimation of inferred costs of these actions and an appropriate choice. Note that choosing to postpone responding for one more time step is also an available action, and has a specific cost associated with it: the cost of opportunity. An action selection *policy* therefore needs to minimize the overall, or expected, cost of action choice inclusive of decision delay costs. These competing goals are made concrete in the form of a *cost function* that specifies the objective to be minimized through the action selection policy. In perceptual decision-making, as an example, a well-studied cost function minimizes a linear sum of response time and accuracy:

(4a) $$Cost=c*RT+c_{e}*P\left(choice\;error\right)+P\left(no\;response\right)$$

The terms in this equation represent the cost of time (parameter c), the cost of choosing the wrong response (*c*_*e*_), and the cost of exceeding the response deadline (which, for simplicity, is normalized to unit cost). P(choice error) and P(no response) are time varying probabilities of making a choice error (due to stimulus misidentification) and making no response, respectively. This sets up a natural *speed-accuracy tradeoff* where the costs of the two available responses depend on the uncertainty of the stimulus identity, and the cost of waiting one more time step may be offset by the possibility of gaining more information. The parameter *c*_*e*_ includes the intrinsic cost associated with error, but may also include extrinsic reward (e.g., the monetary gain/loss received based on the outcome of each trial). Referring back to Figure 1, this cost function forms the basis of estimating action costs based on the current belief state (*b*_*t*_). More specifically, let τ denote the trial termination time, D the response deadline, and d the true stimulus state (e.g., *d* = 0, 1). Then, an action policy π maps each belief state (*b*_*t*_) to a choice of actions (i.e., wait, choose A, or choose B), and over the course of repeated action choices within a trial, results in a termination time τ, and an action choice δ = 0, 1. The loss associated with τ and δ is then:

(4b) $$l(\tau ,\delta ;d,D)=c\tau +c_{e}.1_{\left\{\tau <D,\delta \neq d\right\}}+1_{\left\{\tau =D\right\}}$$

where 1_{·}_ is the indicator function, evaluating to 1 if the conditions in {·} are met and 0 otherwise. Then, on average, the cost incurred by policy π is:

(4c) $$L_{\pi }=<l(\tau ,\delta )>=c<\tau >+c_{e}P(\delta \neq d)+P(\tau =D)$$

where *P*(δ ≠ *d*) is the probability of wrong response, and *P*(τ = *D*) is the probability of not responding before the deadline (omission error). The optimal policy is that policy π which minimizes the average loss, *L*_π_. The modeling work in this domain shows that such an optimal decision policy closely mirrors human and animal behavior in these tasks, in particular, correctly predicting changes in behavior when task constraints are manipulated.

**Figure 3:**
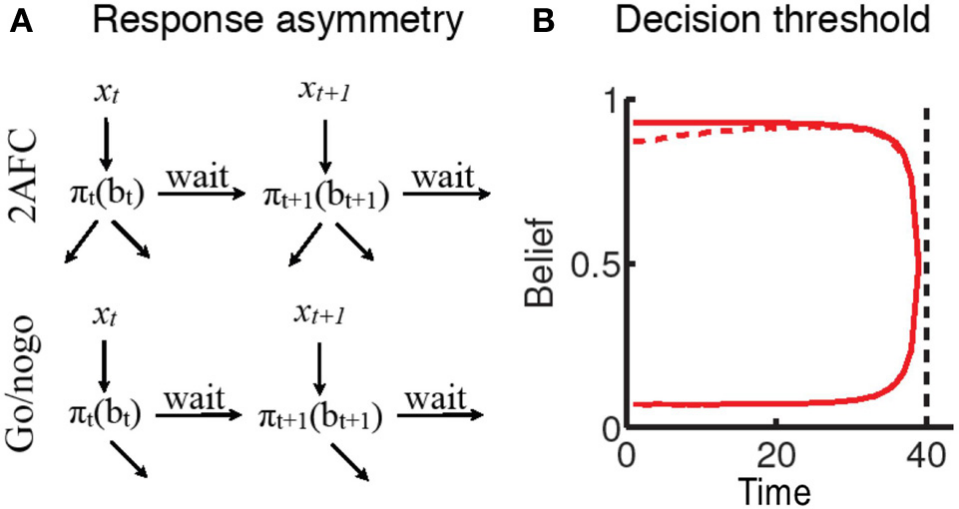
**Rational impatience in the go/nogo task (Shenoy and Yu, 2012). (A)** The rational decision-making framework suggests that choices unfold over time as sensory uncertainty is resolved. For a forced -choice decision-making task, all stimuli eventually result in responses that terminate the trial. For a go/nogo task, the go stimulus requires a go response that terminates the trial; however, the nogo stimulus requires withholding response until the end of the trial; where (*x*_*i*_) and (*y*_*i*_) are the sensory inputs incorporated into beliefs (b_*i*_), and ∏ is the decision policy relating specific beliefs to a choice between actions (a1,…, an, wait). **(B)** The asymmetry is reflected in the decision thresholds for the two tasks: go-nogo response threshold (dashed red line) is initially lower than forced-choice threshold (solid red line), reflecting the tradeoff between go errors and opportunity cost (see text).

One variant of this forced-choice perceptual decision-making task is the 2-alternative forced choice task (2AFC; e.g., Flanker paradigms), in which two stimuli are associated with distinct “go” responses. Another variant is the go/nogo task, where associating one stimulus with an overt response, and the other stimulus with no response during the response window, fundamentally represents a similar perceptual decision process. While on the surface the go/nogo task is very similar to forced-choice decision-making, behavioral and neural evidence suggests an apparent *bias* toward the go response that manifests as a propensity toward high false alarm rates. Such “impatience” has principally been ascribed to failures of putative inhibitory mechanisms (Aron et al., 2007b; Eagle et al., 2008). In contrast, (Shenoy and Yu, 2012) suggest that this behavior may in fact be a rational adaptation of the speed-accuracy tradeoff for this task. To see why this may be the case, consider the schematic representation of the decision-making process in Figure 3. For the 2AFC task, both stimuli eventually lead to a terminating “go” action (one of the two available responses). However, for the go/nogo task, one stimulus leads to a “go” response (and hence termination of the trial), whereas the other stimulus requires waiting until the end of the trial to register a “nogo” response. This asymmetry is reflected in the cost function for the go/nogo task (Shenoy and Yu, 2012):

(5a) $$Cost=c*RT+c_{e}*P\left(false\;alarm\right)+P\left(miss\right)$$

where *c* is the cost of time, *c*_*e*_ is the cost of commission error, *P*(false alarm) and *P*(miss) are the probabilities of making commission and miss errors, respectively.

If again τ denotes the trial termination time and D is the trial deadline, τ = *D* if no “go” response is made before the deadline, and τ < *D* if a response is made. On each trial, the optimum decision policy π has to minimize the following expected loss, *L*_π_:

(5b) $$L_{\pi }=c<\tau >+c_{e}P(\tau <D|d=0)P(d=0)+\;P(\tau =D|d=1)P(d=1)$$

where *P*(*d* = 0) = *P*(NoGo) and *P*(*d* = 1) = *P*(Go) are the probabilities that the current trial is NoGo or Go, respectively, *P*(τ < *D*|*d* = 0) is the probability that a NoGo trial is terminated by a Go response (false alarm), and *P*(τ = *D*|*d* = 1) is the probability that no response is emitted before *D* on a Go trial (miss). Here, *P*(Go) and *P*(NoGo) reflect prior beliefs about the current trial type being a Go or a NoGo trial respectively (i.e., action expectancies), whereas *P*(τ < *D*|*d* = 0) and *P*(τ = *D*|*d* = 1) are the overall fraction of false alarm or miss error respectively. Note that a correct NoGo response consists of a series of “wait” actions until the response deadline D is reached.

Compare this with the previous cost function (Equations 4a–c), for perceptual decision-making. In both tasks, the decision to “go”/terminate the trial (i.e., τ < *D*) limits the costs associated with response delay, and the choice to “wait” (i.e., τ = *D*) decreases error related costs since it results in additional data observation and therefore helps the disambiguation process. Bellman's dynamic programming principle (Bellman, 1952) can be used to determine the optimum decision policy (i.e., smallest expected costs of go vs. wait actions), which is computed iteratively as a function of the belief state *b*_*t*_, i.e., Q-factors Qw(*b*_*t*_) and Qg(*b*_*t*_) for wait and go actions, respectively. That is, if Qw(*b*_*t*_) < Qg(*b*_*t*_), the optimal policy chooses to wait, otherwise it chooses to go (adapted from Shenoy and Yu, 2011, 2012).

In the go/nogo task, however, the cost function directly trades off response times against the go bias, since shorter RT leads to lower overall cost of time, and a lower miss rate, at the cost of an increase in false alarm rate. This is reflected in the *decision boundaries* corresponding to the forced choice and go/nogo tasks (Figure 3B). In the forced-choice task, whenever the belief in stimulus identity crosses one of two symmetric thresholds, a response is generated. This threshold decreases as the response deadline approaches, since beliefs are unlikely to change drastically in the remaining time. In contrast, the go/nogo threshold is an initially *increasing* single threshold, capturing the notion that early on in the trial, an erroneous go response may be preferable to the prospect of waiting until the end of the trial.

### Inhibitory capacity, task context, and emotion

Here, we examined a rational decision-making framework for inhibitory control, where various behavioral effects (and associated measures of inhibitory capacity or failure) were seen as emergent properties of an evolving cost-benefit tradeoff. This view captures behavior in a range of tasks such as the Stroop task, the Eriksen task, the go/nogo task, and the stop signal task, each of which is used to study a putatively different aspect of inhibitory control. Specifically, we described two classes of parameters that capture the dynamic decision-making process supporting inhibitory control, namely those representing (1) individuals' beliefs about task-related events and (2) the relative values associated with these events. In terms of belief estimation, we consider *action expectancies* (e.g., probability of encountering a stop or go trial), as well as *outcome expectancies* (e.g., probability of making an error, of encountering an appetitive stimulus). Similarly, for valuation processes, our model distinguishes action related costs (e.g., time/opportunity or activation costs) and outcome related costs (e.g., cost of error). Summing up the implications of this work, we see that the different behavioral measures of inhibitory capacity are all attributable to one or more specific *constituent parameters* of the decision-making framework which subserves performance in all of these tasks. Thus, seemingly disparate functions such as action, restraint and cancellation, attentional and behavioral inhibition, can be folded into a unifying framework of *information* and *valuation*, where the diversity of behavior principally reflect subtle differences in the task design, and their subsequent influence on components of the model. This perspective guides our view of the potential roles of emotion in inhibitory control: By conceptualizing emotion as *additional context* available to (or imposed upon) the decision-maker, we may then generate constrained hypotheses about how such emotional context may impact behavior within the confines of our proposed decision-making framework. Through this exercise, we aim to relate emotion directly to other, better-understood aspects of cognition such as beliefs, valuation, and choice.

## A bayesian framework for affect-driven biases in inhibitory control

We now examine how the computational framework outlined above can be used to understand emotional influences on inhibitory control. In particular, we hypothesize that each of the primary emotional dimensions considered (i.e., valence/motivational tendency and arousal) may be understood in terms of their biasing effects on parameters formalizing: (a) the values and shape of prior probability distributions, and (b) the relative values of various actions/outcomes. The former focuses on the generative models that guide the inference of beliefs from available evidence (i.e., information acquisition and maintenance), while the later refers to cost functions that constrain the action selection policy (i.e., valuation).

**Figure 4:**
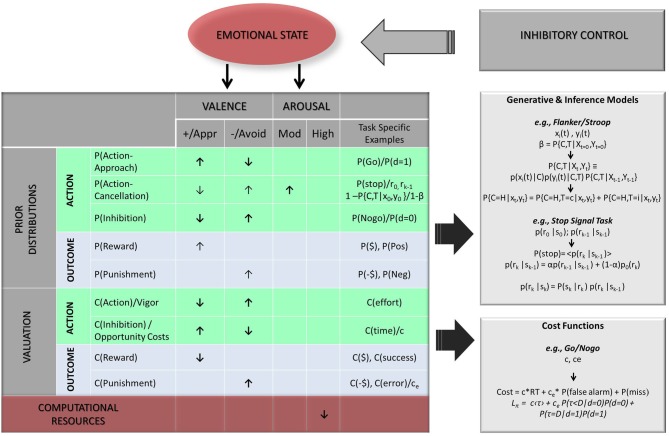
**Hypothesized biases of emotional dimensions on Bayesian model parameters.** Two categories of parameters are considered: prior probability distributions [means; *P*(); top panel] and relative costs [*C*(); bottom panel], each being further evaluated in terms of primary action related expectancies (green areas) and task contingent outcomes (light blue areas). Legend: arrows indicate hypothesized direction of bias, with bolded arrows indicating stronger or more likely biases (↑, increase/higher value; ↓, decrease/lower value); Valence Dimension: +/Appr, positive/approach; −/Avoid, negative/avoidance; Arousal: Mod., moderate; Pos, positive/rewarding outcome/stimulus; Neg, negative/punishing outcome/stimulus; $, monetary reward; -$, monetary penalty; α, mixing factor; *P*(*C, T*|*X*_0_, *Y*_0_)/β, probability of trial being congruent at trial onset *t* = 0 (e.g., in Stroop or Flanker task); *x*(*t*) = sensory input for central stimulus, *y*(*t*) = sensory input for flanker stimulus; *P*(pos), probability of positive stimulus/outcome (e.g., happy face), *P*(Neg), probability of negative stimulus/outcome (e.g., angry face, painful stimulus); *P*(go) = *P*(*d* = 1) = probability of upcoming trial being Go trial; *P*(NoGo) = *P*(*d* = 0) = probability of upcoming trial being Nogo trial, *P*(stop) = probability of upcoming trial (*k*) being Stop trial (*r*_0_ = initialization prior value at first trial; *r*_*k* − 1_, initialization prior value from previous trial); α, mixing coefficient; *P*(τ < *D*|*d* = 0), probability of making “false alarm error” (incorrect go responses), *P*(τ = *D*|*d* = 1) = probability of making “miss” error (incorrect nogo response); *C*(time) = *c*, cost of time, *C*(effort), cost of effort associated with action; *C*(error) = *c*_*e*_, cost of error; τ, trial termination time; *D*, trial deadline; d, true stimulus state (e.g., here *d* = 0 for NoGo trials, *d* = 1 for Go trials).

In this review, we confine ourselves to computational hypotheses within the decision-making framework—i.e., hypotheses about how emotion may be viewed as additional context informing and constraining existing, ongoing computations. We break down emotional influence into valence/motivational tendency and arousal, two empirically validated dimensions of emotion, and consider their potential impact on both action related computations (Figure 4 green areas) and outcome related computations (Figure 4 blue areas). However, we also consider possibilities where emotion processing may act as a separate, *competing process* diverting attentional and executive resources away from task-related computations. As we discuss below, this becomes particularly relevant to the effect of arousal.

### Probabilistic computation

One way to conceptualize the interaction of emotion and inhibitory control within a Bayesian framework relates to sensory disambiguation and belief formation (e.g., expectations about task relevant stimuli/outcomes). We suggest that the values and shape of the prior probability distributions associated with given events are the computational levels where such affective influences could be implemented. Such probabilistic computations represent an individual's prior knowledge of the environment in which he/she is operating, which is used to make predictions about upcoming events. For instance, a central assumption of the Bayesian ideal observer model is an iterative estimation of the likelihood of certain events as sensory disambiguation proceeds until certain probability thresholds that minimize the cost function are reached (at which point an action is selected). These probability distributions may also be updated over the course of multiple trial/response dyads (generating posterior distributions) based on the history of prior estimates and current trial outcome (Bayes rule; e.g., Equation 3b). Thus, prior distributions are often modeled as the combination of a fixed initial prior (representing pre-task frequency estimates) and the previous posterior distributions capturing the history of multiple trials in the task (Shenoy and Yu, 2011); see Equation 3a). While factors such as previous experience with the inhibitory task are likely to heavily influence these prior values, we propose that emotional attributes could be similarly used as heuristics to gauge how likely an event or upcoming action is, resulting in a general shift in values (i.e., mean change) or changes in the distribution shape (e.g., variance, skew; see Figure 4 top panel “Prior Distributions”). Supporting the plausibility of this hypothesis, there is robust evidence of similar biases in subjective probability estimation in healthy populations, typically reflecting underestimation of high probabilities and overestimation of low probabilities (Kahneman and Tversky, 1979; Loewenstein and Lerner, 2003).

Based on the reviewed literature and extensive evidence of interdependence between valence laden information and action tendencies (e.g., activation vs. inhibition; see (Huys et al., 2011; Dayan, 2012), we consider two mediating mechanisms by which valence and arousal could bias probabilistic computations, including outcome expectancies (see Figure 4 top panel, blue area), and action expectancies (see Figure 4 top panel, green area). Finally, given evidence of distinct functional and neurochemical systems involved in approach related actions (e.g., “go”), action cancellation (e.g., stopping an initiated action, “stop”), and inhibition (e.g., withholding an action, “no-go” (Frank, 2005; Eagle et al., 2008; Swick et al., 2011), our proposed model distinguishes these three types of action requirements when considering potential emotional influences. We note that approach-based activation in the context of standard inhibitory paradigms is most commonly associated with go actions, which could be in the context of gaining a reward or avoiding “miss” errors. The latter is more akin to a form of *active avoidance* (i.e., performing an action to avoid a negative outcome). In contrast, inhibition or action restraint in the present framework (e.g., “nogo” responses) is related to *passive avoidance* (e.g., not performing an action to avoid “false alarm” errors or other penalties). This is consistent with actor-critic models of reinforcement learning (Maia, 2010; Dayan, 2012) and neural evidence that learning of both approach actions and avoidant actions involve phasic firing of dopamine neurons (predominantly via D1 receptors) in the dorsal striatum (Montague et al., 2004; Samson et al., 2010). In contrast, dips in dopamine (via D2 receptors in the “no-go” indirect pathway) and serotonin may be primarily involved in mediating inhibition or action constraint (Frank et al., 2004; Dayan and Huys, 2008; Kravitz et al., 2012).

#### Valence/motivational tendency

***Action expectancies.*** The valence of an emotional state provides information about one's disposition toward stimuli or actions in the environment (Schwarz and Clore, 1983), with positive valence promoting approach and negative valence promoting avoidance. Such *motivational* information may in turn be integrated into the interoceptive processes taking place during concurrent inhibitory control behavior. Thus, we suggest that emotion may exert influence on behavior by modulating expectations of encountering specific action requirements (i.e., trial types) relevant to the inhibitory control task. For example, in a go/no-go paradigm, one has to choose between two types of behavioral responses, namely a “go”/approach action or a “no-go”/inhibition response. We hypothesize that positive valence may promote approach actions by increasing expectancies of having to implement an approach action (e.g., expectation to encounter a “go” trial) or decreasing expectancies of implementing action restraint (e.g., “no-go” trial), while negative valence may have the opposite effect. In probabilistic terms, the positive interoceptive information conferred by an emotional state may increase an initial and fixed prior's values (e.g., an overall mean shift of the distribution) for go trials [i.e., *P*(*d* = 1) = *P*(Go)], as they involve an approach action, and/or decrease such prior values for no-go inhibitory trials [i.e., *P*(*d* = 0) = *P*(NoGo) = 1-*P*(Go)]. Either of these biases would promote faster go decisions (and higher rates of false alarm errors) as shorter go reaction times (τ) would minimize the cost function (see Equation 5b). This is because such higher prior over the frequency of go stimuli would provide a higher starting point for the evidence accumulation process, thus requiring a shorter time for the belief state (*b*_*t*_) to reach the decision boundary and generate a go response; see (Shenoy and Yu, 2012). Alternatively, a negative emotional state should have the opposite effect in biasing upward no-go prior values (and/or decreasing go prior values), resulting in longer go reaction times (and more miss errors).

An extensive behavioral and neural literature suggests hedonic valence and action tendencies have strong interdependence, supporting our hypotheses. For instance an appetitive state (e.g., conditioned appetitive cue) promotes approach actions and hinders withdrawal and action constraint/no-go responses, while aversive cues have the reverse effect (Huys et al., 2011; Guitart-Masip et al., 2011b). Individuals are also more likely to learn go actions in rewarded conditions and less likely to learn passive avoidance (i.e., no-go choices) in punished conditions (Guitart-Masip et al., 2011b, 2012). Similarly, higher commission rates are observed when appetitive stimuli are paired with a no-go (i.e., action restraint) requirement (Hare et al., 2005; Schulz et al., 2007; Albert et al., 2011). Here, the positive valence/approach motivation may increase expectations of encountering a go trial [i.e., higher *P*(Go)], again promoting earlier responses (i.e., shorter τ; see Equation 5b). Importantly, valence congruent effects are also observed with the valence of an action (i.e., approach vs. withdrawal). For instance, Huys et al. (2011) showed that even after controlling for behavioral activation/inhibition and the valence of contingent rewards/punishments, an active withdrawal response was facilitated by aversive states but inhibited by an appetitive state. Similarly individuals scoring higher on trait measures of reward expectations demonstrate slower SSRTs, while those with higher punishment expectations produce faster SSRTs in stop-signal tasks (Avila and Parcet, 2001). Thus, while appetitive states may increase go trials expectancies, they may *decrease* expectancies of encountering action cancellation trials [i.e., *P*(stop) = <*p*(*r*_*k*_|*s*_*k* − 1_)> in Equation 3a] while the reverse is true for aversive states.

***Outcome expectancies.*** Consistent with connectionist (or neural network) accounts (Mathews and Macleod, 1994), emotional states have been shown to activate mood-congruent information and concepts in memory, which in turn increases the likelihood this information is attended to (Forgas et al., 1984; Eich et al., 1994; Bower et al., 2001). We suggest that these mood-congruent effects, by modulating the “landscape” of information in awareness, produce biased expectations of encountering valence-congruent outcomes. Again, these biases could manifest by increases or decreases in the central tendency and/or shape of the prior probability distributions associated with valence laden events. For instance, negative affect, such as sadness and anxiety, promotes higher expectations of punishment and aversive events (Abramson et al., 1989; Ahrens and Haaga, 1993; Handley et al., 2004), while euphoria is associated with higher expectations or reward and success (Johnson, 2005; Abler et al., 2007). In addition, relative to euthymic controls, sad or depressed individuals are more accurate and faster at recognizing sad affect in human faces (Lennox et al., 2004), while socially anxious individuals are better at identifying angry faces (Joormann and Gotlib, 2006). In contrast, manic individuals are less accurate at identifying sad faces (Lennox et al., 2004). These biases have been linked to different neural patterns in face recognition areas, suggesting a different prior “expertise,” rather than differences in emotional response. In the context of action requirements tied to emotional cues (e.g., in affective go no-go paradigms), such biases would result in a reduced discrepancy between internal predictions of encountering a mood congruent stimulus [e.g., positive or negative facial expression, i.e., *P*(Pos)/*P*(Neg) Figure 4] and the actual occurrence of this event. This should in turn facilitate (i.e., speed up) the identification of mood-congruent stimuli in emotional relative to euthymic individuals. Consistent with this hypothesis, in affective go no-go paradigms, manic patients respond faster to happy stimuli and slower to negative stimuli on go trials, and depressed patients respond faster to sad stimuli (Murphy et al., 1999; Erickson et al., 2005; Ladouceur et al., 2006). These types of emotional biases could impact inhibitory function more indirectly than those associated with action requirement expectancies, possibly by facilitating or slowing the disambiguation of emotional cues tied to action requirements. This may be particularly relevant for inhibitory control within social interactive contexts.

#### Arousal

***Action expectancies.*** Increased arousal has been associated with impaired functioning of the prefrontal cortex (PFC), including regions necessary to implement inhibitory control such as the inferior frontal gyrus (Robbins and Arnsten, 2009). In addition, high arousal promotes stronger reliance on habitual/prepotent responses and generally decreases goal-directed responding (Dias-Ferreira et al., 2009; Schwabe and Wolf, 2009). Therefore, we suggest that high arousal is more likely to impair inhibitory control by reducing the attentional and computational resources necessary to disambiguate task relevant information (see Figure 4; red area). This is consistent with studies linking arousal prompted by conditioned cues to electric shock to a selective slowing during inhibitory trials in Stroop and Stop-signal tasks (Pallak et al., 1975; Pessoa et al., 2012). Indeed, because incongruent, non-prepotent, responses involve more sensory disambiguation and/or more effort to shift response set, such computational processes may more heavily rely on intact PFC function and executive resources. Therefore, the taxing of PFC function under high arousal would be expected to more selectively impact performance during incongruent trials. Other work, however, points to a more general arousal-driven impairment for both prepotent and inhibitory responses, notably in Stroop (Blair et al., 2007), stop-signal (Verbruggen and De Houwer, 2007), and go/no-go (De Houwer and Tibboel, 2010) paradigms.

In contrast, evidence suggests that *moderate* levels of arousal can facilitate executive and PFC function, consistent with an inverted U shape relationship between arousal and cognitive performance (Easterbrook, 1959; Eysenck, 1982; Arnsten, 1998). Moderate levels of norepinephrine (NE) release strengthen prefrontal cortical functions via actions at post-synaptic α-2A adrenoceptors with high affinity for NE, which has been associated with improved set shifting function and selective attention (Ramos and Arnsten, 2007). Based on this literature, we propose that moderate arousal may facilitate activation, particularly action cancellation (e.g., stop response), by increasing expectancy of encountering stop trials. This is consistent with extensive animal literature highlighting the role of NE as a neural “interrupt” (Sara and Segal, 1991; Dayan and Yu, 2006) and recent studies showing that both NE and dopamine play an important role in regulating impulsivity and speed of behavioral control in ADHD (Arnsten et al., 1984; Frank et al., 2006; Arnsten, 2009b). Consistent with this hypothesis, both human and animal studies point to a selective facilitating effect of norepinephrine in stop-signal paradigms, improving SSRTs while go reaction times are typically unchanged (Overtoom et al., 2003; Chamberlain et al., 2006; Robinson et al., 2007). Moderate arousal induced by *both* positive and aversive images were also found to improve SSRTs in humans (Pessoa et al., 2012). This contrasts with pharmacological studies that suggest no effect of dopamine or serotonin on SSRTs, but rather preferential effects on go/approach actions and action constraint/inhibition respectively (Eagle et al., 2008). Computationally, moderate arousal may increase the mean of the prior distribution associated with the frequency estimate of stop trials *p*_0_(*r*_*k*_), which in turn would result in a similar upward shift in the predictive probability of stop trials *P*(stop) [i.e., the mean of the predictive distribution *p*(*r*_*k*_|***s***_*k* − 1_), see Equations 3a and b].

In relation to action cancellation, arousal should similarly bias expectancies related to cancelling automated responses in interference paradigms (e.g., interruption of prepotent responses during incongruent trials in Stroop or Flanker tasks). Specifically, moderate arousal may increase expectations of encountering incongruent events (requiring action cancellation) or decrease expectations of encountering congruent trials, which would result in less impairment in incongruent/inhibitory trials. For instance, in flanker paradigms (and presumably in other interference tasks), modeling the sensory disambiguation process with a joint probability of true stimulus and trial type [i.e., *P*(*C, T*|*X*_*t*_, *Y*_*t*_), see Equation 1] produces inferential performance that successfully captures behavioral data. Importantly, increasing its initialization prior to reflect a bias toward compatibility [*P*(*C, T*|*X*_0_, *Y*_0_), β > 0.5/chance] produces a shift in inference that would be expected to lead to worse performance on incompatible trials (Yu et al., 2009). This relates to a longer latency for the probability of the trial being incongruent to rise up toward 1 on incongruent trials (as it starts from a lower anchor value). Thus, while such compatibility bias is observed in normative samples (Yu et al., 2009), we hypothesize that moderate arousal could reduce this bias, which would be reflected by a lower value of the β parameter in the model (i.e., closer to 0.5). This is consistent with improved Stroop performance in moderate arousal condition (mild shock expectation; Pallak et al., 1975).

***Outcome expectancies.*** While we are not aware of any studies isolating the effect of arousal from valence on outcome expectations, some work suggests that prolonged physiological arousal in anxiety and trauma conditions may play a role in maintaining expectations of danger (Norton and Asmundson, 2004). It remains difficult, however, to disentangle the role of arousal from valence in these effects, which may be better explained by valence-congruent effects on memory and attention (see above). Thus, we suggest that the arousal dimension is unlikely to impact outcome expectancies (e.g., reward vs. punishment), but rather modulates action preparedness and expectations of encountering action cancellation trials (via NE release as previously noted). Indeed, based on the affective go/no-go studies, *valence*-congruent response biases in go reaction times were observed in depressed and manic patients (Murphy et al., 1999; Ladouceur et al., 2006) as opposed to a unidirectional effect of emotion (which would be more consistent with an arousal effect). This speaks against a potential role of moderate arousal in biasing probabilistic outcome expectancies. In addition, higher levels of arousal are likely to have a deleterious impact on computational recourses mediated by impaired PFC function (Ramos and Arnsten, 2007).

#### Neural implementation

Valence-dependent biases on approach activation and inhibition tendencies are likely to preferentially involve the dopamine and serotonin signaling in the dorsal striatum. The approach “go” pathway is facilitated by positive/rewarding states via dopamine (D1 receptors) while serotonin and dopamine (D2 receptors) are preferentially involved in linking negative/aversive valence to the inhibition/”nogo pathway (Frank et al., 2004; Montague et al., 2004; Dayan and Huys, 2008). Active withdrawal and action cancelation may also involve serotonin (Deakin and Graeff, 1991). In addition, norepinephrine and dopamine are likely to play a key role in mediating arousal effects on action cancelation by facilitating fronto-striatal communication (Ramos and Arnsten, 2007; Eagle et al., 2008). In terms of brain regions, probability computation (in contrast to valuation) within an expected utility framework has been associated with activation of the mesial PFC (Knutson and Peterson, 2005), although recent evidence points to subcortical correlates in anterior and lateral foci of the ventral striatum (Yacubian et al., 2007). While this is still an emerging program of research, recent work also suggests that the dorsomedial PFC encodes in a dose-response manner a representation of the history of successive incongruent trials in interference paradigms (Horga et al., 2011). Such neural representations appear critical to maintaining cognitive control in the task, as they influenced the neural and behavioral adaptation to incongruency in this task supported by a network involving the pre supplementary motor areas (SMA) and dorsal anterior cingulate (dACC). Based on this research, computational biases related to the cumulative magnitude of certain event probabilities (e.g., expectancy of action cancellation requirement), including those driven by emotion, may be reflected by differential recruitment of the dorsomedial PFC. In addition, converging evidence suggests that the dACC is involved in tracking conflict (Botvinick et al., 1999, 2001) and more generally expectancy violation (Somerville et al., 2006; Kross et al., 2007; Chang and Sanfey, 2011). In line with a conflict monitoring hypothesis, activation of this region is indeed consistently observed during incongruent/high conflict trials in various inhibitory control tasks (Botvinick et al., 2001) and predicts subsequent prefrontal recruitment and behavioral adjustments (Kerns et al., 2004; Kerns, 2006). Importantly, recent computational work highlights the selective involvement of the dACC in coding the discrepancy between internally computed probabilities of response inhibition and actual outcome, a form of “Bayesian prediction error” (Ide et al., 2013), making this region a plausible candidate for tracking the magnitude of potential emotion-driven biases in Bayesian error prediction.

### Valuation

We now consider emotion-driven biases associated with valuation processes and argue that emotional attributes may increase or decrease the relative costs of task-related actions and outcomes. Based on extensive empirical and computational evidence from the reinforcement learning literature, a representation of the values (or expected reward) associated with possible actions is necessary to support the selection of actions in goal-directed behavior (Montague et al., 2006). Mesolimbic dopamine has been posited to play a crucial role in the “binding” of such hedonic values and reward-related actions or stimuli, providing a motivational weight or “incentive salience” to these actions/stimuli (Berridge and Robinson, 1998; Berridge, 2007). Thus, as with any type of goal-directed behavior, the selection of actions involved in inhibitory control tasks (e.g., go vs. no-go actions) should be modulated by such a valuation system. Consistent with this hypothesis, manipulating the perceived value of response speed vs. accuracy (e.g., with subtle changes in instructions) produces behavioral changes in concert with the expected motivational shifts in stop-signal paradigms (Band et al., 2003; Liddle et al., 2009). Overall this suggests that the relative values associated with task-related actions/events contribute to modulating inhibitory behavior independently of probabilistic computations (e.g., action requirement expectancies). Because emotion again conveys information about one's state and disposition (Schwarz and Clore, 1983), an intuitive prediction is that the *valence* of an emotional state is likely to modulate the incentive salience (i.e., value) of particular task-related actions/outcomes. In Bayesian terms, the relative weight or salience of these actions/events is reflected in the cost function, and most commonly in terms of speed vs. accuracy tradeoffs (see Equations 4a, 5a). As with the probabilistic computation section, we consider valuation biases separately for task-related actions (e.g., go vs. no-go; Figure 4, bottom panel, green area) and outcomes (e.g., accuracy; Figure 4, bottom panel, blue area). Based on limited evidence for distinct valuation mechanisms for different types of action requirements, and given previous work linking reward with the degree of effort/vigor of a particular action (Niv et al., 2007), we simplify the action category to basic (approach-based) activation and inhibition.

#### Valence/motivational tendency

***Action valuation.*** Some animal studies suggest that phasic release of dopamine in the NAcc is involved in coding the predictive reward of an action and is directly related to the degree an animal overcomes and maintains effort to obtain this reward (Morris et al., 2006; Phillips et al., 2007; Salamone et al., 2007). This research points to a potential role of NAcc dopamine in representing effort-related costs (i.e., associated with behavioral activation). In a closely related line of work, recent computational accounts suggest that tonic levels of dopamine release encode the average rate of available reward per unit of time, which is inversely proportional to *opportunity costs* associated with slower responses (Niv et al., 2007; Shadmehr, 2010; Guitart-Masip et al., 2011a). In contrast to those associated with effort (i.e., activation), opportunity costs can be conceptualized as cost of time or “waiting to act” (i.e., inhibition).

Based on this research, we conjecture that the degree to which an emotion is appetitive may modulate the value of engaging in action (e.g., reducing the cost of effort associated with behavioral responses). For instance, in affective go/no-go paradigms, a positive emotional state (or the anticipation of such state) should reduce the cost of effort associated with go actions [or increase opportunity costs associated with inhibition; i.e., *C*(time) = *c* in Equations 4a–c, 5a–b and Figure 4]. Computationally, either biases should result in selecting go actions at earlier stages of the sensory disambiguation process (i.e., faster reaction times would minimize cost). Similarly, the aversive tone of an emotional state may have the opposite effect, i.e., increasing activation/effort costs, thus promoting inaction. Consistent with these predictions, appetitive Pavlovian stimuli specifically promote “go” actions and inhibit no-action and withdrawal, while aversive cues promote the opposite pattern (Hare et al., 2005; Huys et al., 2011; Guitart-Masip et al., 2012). Importantly, activations in the striatum (ventral putamen) and ventral tegmental area (VTA) have been found to correlate with the magnitude of go and no-go action values with opposite signs for each respective action (Guitart-Masip et al., 2012).

***Outcome valuation.*** Appetitive vs. aversive emotional states can have valence-congruent modulating effects on hedonic experience. For instance a depressed or sad mood reduces the pleasantness of rewards and amplifies perception of pain, while positive mood lowers pain ratings and increases pain tolerance (Tang et al., 2008; Zhao and Chen, 2009; Berna et al., 2010). This is consistent with extensive evidence that negative mood states are associated with reduced sensitivity to reward (Henriques and Davidson, 2000; Harlé and Sanfey, 2007; Foti and Hajcak, 2010; Disner et al., 2011), as well as increased sensitivity to error (an aversive event) demonstrated by stronger amplitudes of the error related negativity (ERN) (Paulus et al., 2004; Pizzagalli et al., 2005; Olvet and Hajcak, 2008; Wiswede et al., 2009; Weinberg et al., 2010) and more post error slowing (Luu et al., 2000; Boksem et al., 2008; Compton et al., 2008). In contrast, appetitive states have been linked to increased reward sensitivity (Johnson, 2005), increased perception of happiness (Trevisani et al., 2008) and reduced post error slowing in interference tasks, consistent with a reduced monitoring of error (van Steenbergen et al., 2009, 2010).

Accordingly, we suggest that, in addition to modulating action valuation, the valence of an emotional state may bias the relative value/cost of task-related outcomes (e.g., rewards and punishments associated with performance). Specifically, positive emotion should enhance the relative value, i.e., decrease the relative cost of rewarding outcomes [e.g., *C*($) Figure 4]. In contrast, negative emotional states would be more likely to prompt an overestimation of the cost of error or other aversive events [i.e., *C*(-$), *C*(error)/*c*_*e*_, see Equations 4a–c, 5a–b and Figure 4)]. For instance, to minimize average costs in a go/no-go task (see Equations 5b), this over-weighing of false alarm costs (i.e., higher value of *c*_*e*_) would be associated with a lower threshold for the rate of false alarm occurrence across trials [i.e., *P*(false alarm) = *P*(τ < *D*|*d* = 0) *P*(*d* = 0) = *P*(τ < *D*|*d* = 0) *P* (NoGo); see Equation 5a,b). This would in turn prompt longer response times needed for sensory disambiguation to unfold and for *P*(NoGo) to reach a lower threshold. This is because the cost associated with go actions [Qg(*b*_*t*_)] would be overall higher, requiring more time to drop lower than the cost of waiting [Qw(*b*_*t*_)]. Although we are not aware of any study specifically testing this relationship, depressed individuals were slower on go trials and made less commission errors in a parametric go no-go paradigm, suggestive of heightened concern for errors (Langenecker et al., 2007). Similarly, in individuals with generalized anxiety disorder, better performance on a classic color-word Stroop has been linked to higher levels of worry and trait anxiety (Price and Mohlman, 2007).

#### Arousal

***Action valuation.*** The clinical and social psychology literature suggest that physiologically induced arousal can be misattributed in evaluative processes such as interpersonal preferences and risk assessment (Schachter and Singer, 1962; Sinclair et al., 1994). This is reflected by more extreme intensity ratings of either positive or negative stimuli, suggesting a *unidirectional* (i.e., enhancing) role of arousal in modulating hedonic ratings of *concurrent* events. For instance, perceived arousal in the context of positive stimuli leads to higher positive valence ratings, while increased arousal in a negative context leads to higher aversive ratings (Storbeck and Clore, 2008). Thus, rather than arousal independently modulating valuation processes, it is the interaction of arousal and valence which seems to produce valuation biases. This fits with the neural and physiological literature highlighting the role of arousal in modulating *attention* to particular stimuli and action preparedness (Schutter et al., 2008; Gur et al., 2009), hence our proposal it may contribute to probabilistic expectancy biases (see section Probabilistic Computation). Based on this literature, we suggest this generally speaks against an independent effect of arousal on valuation processes.

***Outcome valuation.*** As mentioned above, arousal may play a “magnifying” role in valuation processes by interacting with appetitive or aversive valence. This could argue for arousal promoting unidirectional increase in the relative weights of valence-laden computational elements in the cost function. That is, the value of both positive and negative task-related outcomes, such as performance dependent rewards [i.e., *C*($)] and penalties [i.e., *C*(-$) see Figure 4] would be increased. Arousal in the context of punishment sensitivity in anxiety may further increase the relative weight of error in the cost function (e.g., *c*_*e*_ in Equations 5a,b), which would in turn lead to slower responses (to minimize overall costs) and possibly decreased error rates. This is consistent with the positive relationship observed between worry/anxious preoccupation and reaction times in anxious individuals (Price and Mohlman, 2007). However, in this study, reaction times were *not* correlated with anxious arousal *per se*, which makes these results more consistent with valence dependent biases (see above). In addition, while higher levels of arousal have been associated with a general slowing in euthymic individuals independently of positive vs. negative emotional context (Blair et al., 2007; Verbruggen and De Houwer, 2007; Pessoa et al., 2012), this pattern may again be more parsimoniously explained by an impairment of PFC function and related depletion of attentional and executive resources (Arnsten, 2009a).

#### Neural implementation

At the neural level, the ventral striatum (specifically the nucleus accumbens) has been consistently associated with reward sensitivity and reward based learning; (Knutson et al., 2001; O'Doherty, 2004; Winkielman et al., 2007). An important body of research has shown that phasic release of dopamine in the NAcc is involved in learning the predictive value of conditioned stimuli (Schultz et al., 1997; Flagel et al., 2010), which is thus likely to play a role in the coding of task related outcomes and stimuli (e.g., response cues, error or reward contingent on performance). Other research further suggests that tonic dopamine levels in this region is involved in coding opportunity costs associated with waiting to act (Niv et al., 2007; Shadmehr, 2010), while phasic dopamine release may be involved in the representation of effort associated with goal directed behavior (Phillips et al., 2007; Salamone et al., 2007). This is consistent with findings of caudate activation during inhibition (no-go responses) in positive/appetitive context, which was proportional to commission error rates (Hare et al., 2005). Finally recent computational work has identified areas in the ventral striatum and VTA as specifically encoding instrumentally learnt values of go and no/go actions (Guitart-Masip et al., 2012). These regions are therefore plausible neural markers for tracking action valuation biases. In addition, activation of the anterior insula has been associated with sensitivity to monetary losses (a punishing outcome) and learning from aversive outcomes (Kuhnen and Knutson, 2005; Paulus et al., 2005; Samanez-Larkin et al., 2008) including in the context of a negative mood state (Harlé et al., 2012). Thus, valuation biases related to aversive states and punishment expectancy may involve this region. Finally, given its implication in reward valuation (O'Doherty, 2004; Montague et al., 2006) and in integrating motivational attributes of various stimuli into decision-making [somatic markers; see (Damasio, 1994)], the OFC is likely to be involved in the integration of emotional context in valuation biases.

## Summary

We described a simple, unifying framework for inhibitory control that serves as a comprehensive scaffold to integrate emotional influences on cognitive processes. In our view, emotion can be understood as additional context (e.g., interoceptive experience), which constrains and biases the computations in an “ideal observer model” of inhibitory control. That is, the role of affect in inhibitory control can be interpreted in terms of well-understood computational aspects of cognition such as beliefs, action valuation and choice. Thus, emotion may affect inhibitory behavior by biasing (a) prior expectations and associated changes in internal beliefs about various task-relevant events, and (b) action/outcome valuation (see Figure 4). Importantly, on the basis of behavioral and neural data, the framework highlights a strong interdependence between the appetitive/aversive nature of emotional states and basic action tendencies that are intrinsic to inhibitory control. Thus, we surmise that the valence dimension may have primary influences on action parameters associated with approach and inhibition (action constraint), and exert valence congruent influences on outcome valuation and expectancies. In contrast, arousal may have a more selective role in biasing expectancies of action cancellation. In addition, we argue that higher levels of arousal may more indirectly modulate the computational processes supporting inhibitory function by redirecting attention away from task-relevant information and generally impairing prefrontal function and related computational mechanisms. Our theoretical framework has some limitations inherent to the challenge of testing these hypotheses. For instance, the separate effect of valence and arousal are difficult to disentangle in both experimental settings and affective disorders. The breadth of individual variability in the experience and regulation of emotion make these potential effects further difficult to pinpoint.

With regard to the potential impact of emotion on sensory disambiguation, we have emphasized the contribution of outcome and action expectancies (i.e., prior distributions associated with valence congruent events and trial type). However, we should note that more downstream effects of emotion have been documented. For instance, valence and arousal have been shown to modulate visual processing style (i.e., global vs. detail) and selective attention (e.g., breadth of attentional focus; (Loftus et al., 1987; Basso et al., 1996; Gasper and Clore, 2002). Although outside the scope of this review, modeling potential biases in sensory input parameters (e.g., sensory input mixing factors) may capture additional aspects of the interaction between emotion and inhibitory control.

Finally, an equally important aspect of such emotion-cognition interactions is the iterative nature of any emotion-cognitions interactions. That is behavioral performance and the dynamic feedback received when engaged in inhibitory control tasks are likely to modulate emotional state. As a consequence, the nature and types of biases impacting inhibitory control are likely to emerge from the dynamic interaction between Bayesian computation of response costs, selection of actions, and reception of outcomes, which subsequently affect the Bayesian updating of beliefs. These dynamic processes might be particularly relevant in psychopathological conditions, which emerge over longer periods of time.

## Concluding remarks

A Bayesian computational framework provides a fine-grained quantification of emotion and cognitive control interactions by dividing the observed behavior into several contributing neuro-cognitive subprocesses. This in turn provides a powerful tool to test independent affect infusion hypotheses, which are better able to delineate the complex nature of emotion and psychopathology, and may help refine neurocognitive models of various clinical conditions. For instance, behavioral performance could be used to infer specific quantitative biases in one's cost or reward functions or in one's ability to estimate probability. This approach could shed light on the heterogeneous nature of conditions such as depression or substance dependence, by mapping different subtype profiles to specific computational processes and associated neural markers (e.g., anhedonia, uncertainty avoidance, impulsiveness). Ultimately, this may help refine our understanding of how specific behavioral and pharmacological treatments might address these various biases and thus refine our tailoring and effectiveness of psychiatric treatment.

### Conflict of interest statement

The authors declare that the research was conducted in the absence of any commercial or financial relationships that could be construed as a potential conflict of interest.

## References

[B1] Abler B. Greenhouse I. Ongur D. Walter H. Heckers S. (2007). Abnormal reward system activation in mania. Neuropsychopharmacology 33, 2217–2227 10.1038/sj.npp.1301620 17987058 PMC2574988

[B2] Abramson L. Y. Metalsky G. I. Alloy L. B. (1989). Hopelessness depression: a theory-based subtype of depression. Psychol. Rev. 96, 358–372 10.1037/0033-295X.96.2.358

[B3] Ahrens A. H. Haaga D. A. (1993). The specificity of attributional style and expectations to positive and negative affectivity, depression, and anxiety. Cogn. Ther. Res. 17, 83–98 10.1007/BF01172742

[B4] Albert J. Lopez-Martin S. Tapia M. Montoya D. Carretie L. (2011). The role of the anterior cingulate cortex in emotional response inhibition. Hum. Brain Mapp. 33, 2147–2160 10.1002/hbm.21347 21901794 PMC6870140

[B5] Arnsten A. F. (1998). Catecholamine modulation of prefrontal cortical cognitive function. Trends Cogn. Sci. 2, 436–447 10.1016/S1364-6613(98)01240-6 21227275

[B6] Arnsten A. F. (2009a). Stress signalling pathways that impair prefrontal cortex structure and function. Nat. Rev. Neurosci. 10, 410–422 10.1038/nrn2648 19455173 PMC2907136

[B7] Arnsten A. F. (2009b). Toward a new understanding of attention-deficit hyperactivity disorder pathophysiology. CNS Drugs 23, 33–41 10.2165/00023210-200923000-00005 19621976

[B8] Arnsten A. Neville H. Hillyard S. Janowsky D. Segal D. (1984). Naloxone increases electrophysiological measures of selective information processing in humans. J. Neurosci. 4, 2912–2919 6502211 10.1523/JNEUROSCI.04-12-02912.1984 PMC6564868

[B9] Aron A. R. Behrens T. E. Smith S. Frank M. J. Poldrack R. A. (2007a). Triangulating a cognitive control network using diffusion-weighted magnetic resonance imaging (MRI) and functional MRI. J. Neurosci. 27, 3743–3752 10.1523/JNEUROSCI.0519-07.2007 17409238 PMC6672420

[B10] Aron A. R. Durston S. Eagle D. M. Logan G. D. Stinear C. M. Stuphorn V. (2007b). Converging evidence for a fronto-basal-ganglia network for inhibitory control of action and cognition. J. Neurosci. 27, 11860–11864 10.1523/JNEUROSCI.3644-07.2007 17978025 PMC6673355

[B11] Aron A. R. Robbins T. W. Poldrack R. A. (2004). Inhibition and the right inferior frontal cortex. Trends Cogn. Sci. 8, 170–177 10.1016/j.tics.2004.02.010 15050513

[B12] Avila C. Parcet M. A. (2001). Personality and inhibitory deficits in the stop-signal task: the mediating role of Gray's anxiety and impulsivity. Pers. Individ. Dif. 31, 975–986 10.1016/S0191-8869(00)00199-9

[B13] Band G. P. van der Molen M. W. Logan G. D. (2003). Horse-race model simulations of the stop-signal procedure. Acta Psychol. 112, 105–142 10.1016/S0001-6918(02)00079-3 12521663

[B14] Barch D. M. Braver T. S. Sabb F. W. Noll D. C. (2000). Anterior cingulate and the monitoring of response conflict: evidence from an fMRI study of overt verb generation. J. Cogn. Neurosci. 12, 298–309 10.1162/089892900562110 10771413

[B15] Basso M. R. Schefft B. K. Ris M. D. Dember W. N. (1996). Mood and global-local visual processing. J. Int. Neuropsychol. Soc. 2, 249–255 10.1017/S1355617700001193 9375191

[B16] Behrens T. E. J. Woolrich M. W. Walton M. E. Rushworth M. F. S. (2007). Learning the value of information in an uncertain world. Nat. Neurosci. 10, 1214–1221 10.1038/nn1954 17676057

[B17] Bellman R. (1952). On the theory of dynamic programming. Proc. Natl. Acad. Sci. U.S.A. 38, 716 16589166 10.1073/pnas.38.8.716 PMC1063639

[B18] Berna C. Leknes S. Holmes E. A. Edwards R. R. Goodwin G. M. Tracey I. (2010). Induction of depressed mood disrupts emotion regulation neurocircuitry and enhances pain unpleasantness. Biol. Psychiatry 67, 1083–1090 10.1016/j.biopsych.2010.01.014 20303069

[B19] Berridge K. C. (2007). The debate over dopamine's role in reward: the case for incentive salience. Psychopharmacology 191, 391–431 10.1007/s00213-006-0578-x 17072591

[B20] Berridge K. C. Robinson T. E. (1998). What is the role of dopamine in reward: hedonic impact, reward learning, or incentive salience? Brain Res. Rev. 28, 309–369 10.1016/S0165-0173(98)00019-8 9858756

[B21] Blair K. S. Smith B. W. Mitchell D. G. Morton J. Vythilingam M. Pessoa L. (2007). Modulation of emotion by cognition and cognition by emotion. Neuroimage 35, 430–440 10.1016/j.neuroimage.2006.11.048 17239620 PMC1862681

[B22] Boksem M. A. S. Tops M. Kostermans E. De Cremer D. (2008). Sensitivity to punishment and reward omission: evidence from error-related ERP components. Biol. Psychol. 79, 185–192 10.1016/j.biopsycho.2008.04.010 18571302

[B23] Botvinick M. Nystrom L. E. Fissell K. Carter C. S. Cohen J. D. (1999). Conflict monitoring versus selection-for-action in anterior cingulate cortex. Nature 402, 179–181 10.1038/46035 10647008

[B24] Botvinick M. M. (2007). Conflict monitoring and decision making: reconciling two perspectives on anterior cingulate function. Cogn. Affect. Behav. Neurosci. 7, 356–366 10.3758/CABN.7.4.356 18189009

[B25] Botvinick M. M. Braver T. S. Barch D. M. Carter C. S. Cohen J. D. (2001). Conflict monitoring and cognitive control. Psychol. Rev. 108, 624 10.1037/0033-295X.108.3.624 11488380

[B26] Bower G. H. (1981). Mood and memory. Am. Psychol. 36, 129 7224324 10.1037//0003-066x.36.2.129

[B27] Bower G. H. Forgas J. P. Forgas J. (2001). Mood and social memory. Handb. Affect Soc. Cogn. 95–120

[B28] Breiter H. C. Aharon I. Kahneman D. Dale A. Shizgal P. (2001). Functional imaging of neural responses to expectancy and experience of monetary gains and losses. Neuron 30, 619–639 10.1016/S0896-6273(01)00303-8 11395019

[B29] Brown J. W. Braver T. S. (2005). Learned predictions of error likelihood in the anterior cingulate cortex. Science 307, 1118–1121 10.1126/science.1105783 15718473

[B30] Chamberlain S. R. Muller U. Blackwell A. D. Clark L. Robbins T. W. Sahakian B. J. (2006). Neurochemical modulation of response inhibition and probabilistic learning in humans. Sci. Signal. 311, 861 10.1126/science.1121218 16469930 PMC1867315

[B31] Chang L. J. Sanfey A. G. (2011). Great expectations: neural computations underlying the use of social norms in decision-making. Soc. Cogn. Affect. Neurosci. 8, 277–284 10.1093/scan/nsr094 22198968 PMC3594719

[B32] Compton R. J. Lin M. Vargas G. Carp J. Fineman S. L. Quandt L. C. (2008). Error detection and posterror behavior in depressed undergraduates. Emotion 8, 58 10.1037/1528-3542.8.1.58 18266516

[B33] Craig A. D. (2002). How do you feel? Interoception: the sense of the physiological condition of the body. Nat. Rev. Neurosci. 3, 655–666 12154366 10.1038/nrn894

[B34] Damasio A. R. (1994). Descartes and Error. New York, NY: Putnam

[B35] Davidson R. J. (2003). Affective neuroscience and psychophysiology: toward a synthesis. Psychophysiology 40, 655–665 10.1111/1469-8986.00067 14696720

[B36] Dayan P. (2012). Instrumental vigour in punishment and reward. Eur. J. Neurosci. 35, 1152–1168 10.1111/j.1460-9568.2012.08026.x 22487044

[B37] Dayan P. Huys Q. J. (2008). Serotonin, inhibition, and negative mood. PLoS Comput. Biol. 4:e4 10.1371/journal.pcbi.0040004 18248087 PMC2222921

[B38] Dayan P. Yu A. J. (2006). Phasic norepinephrine: a neural interrupt signal for unexpected events. Network 17, 335–350 10.1080/09548980601004024 17162459

[B39] De Houwer J. Tibboel H. (2010). Stop what you are not doing! Emotional pictures interfere with the task not to respond. Psychon. Bull. Rev. 17, 699–703 10.3758/PBR.17.5.699 21037169

[B40] Deakin J. W. Graeff F. G. (1991). 5-HT and mechanisms of defence. J. Psychopharmacol. 5, 305–315 10.1177/026988119100500414 22282829

[B41] Dias-Ferreira E. Sousa J. C. Melo I. Morgado P. Mesquita A. R. Cerqueira J. J. (2009). Chronic stress causes frontostriatal reorganization and affects decision-making. Science 325, 621–625 10.1126/science.1171203 19644122

[B42] Disner S. G. Beevers C. G. Haigh E. A. P. Beck A. T. (2011). Neural mechanisms of the cognitive model of depression. Nat. Rev. Neurosci. 12, 467–477 10.1038/nrn3027 21731066

[B43] Eagle D. M. Bari A. Robbins T. W. (2008). The neuropsychopharmacology of action inhibition: cross-species translation of the stop-signal and go/no-go tasks. Psychopharmacology 199, 439–456 10.1007/s00213-008-1127-6 18542931

[B44] Easterbrook J. A. (1959). The effect of emotion on cue utilization and the organization of behaviour. Psychol. Rev. 66, 183–201 10.1037/h0047707 13658305

[B45] Eich E. Macaulay D. Ryan L. (1994). Mood dependent memory for events of the personal past. J. Exp. Psychol. Gen. 123, 201–215 10.1037/0096-3445.123.2.201 8014613

[B46] Erickson K. Drevets W. C. Clark L. Cannon D. M. Bain E. E. Zarate C. A. (2005). Mood-congruent bias in affective go/no-go performance of unmedicated patients with major depressive disorder. Am. J. Psychiatry 162, 2171–2173 10.1176/appi.ajp.162.11.2171 16263859

[B47] Eysenck M. W. (1982). Attention and Arousal: Cognition and Performance. New York, NY: Springer-Verlag 10.1007/978-3-642-68390-9

[B48] Flagel S. B. Clark J. J. Robinson T. E. Mayo L. Czuj A. Willuhn I. (2010). A selective role for dopamine in stimulus-reward learning. Nature 469, 53–57 10.1038/nature09588 21150898 PMC3058375

[B49] Forgas J. P. (2002). Feeling and doing: affective influences on interpersonal behavior. Psychol. Inq. 13, 1–28 10.1207/S15327965PLI1301_01

[B50] Forgas J. P. Bower G. H. Krantz S. E. (1984). The influence of mood on perceptions of social interactions. J. Exp. Soc. Psychol. 20, 497–513 10.1016/0022-1031(84)90040-4

[B51] Foti D. Hajcak G. (2010). State sadness reduces neural sensitivity to nonrewards versus rewards. Neuroreport 21, 143–147 10.1097/WNR.0b013e3283356448 20010444

[B52] Frank M. J. (2005). Dynamic dopamine modulation in the basal ganglia: a neurocomputational account of cognitive deficits in medicated and nonmedicated Parkinsonism. J. Cogn. Neurosci. 17, 51–72 10.1162/0898929052880093 15701239

[B53] Frank M. J. Santamaria A. O'Reilly R. C. Willcutt E. (2006). Testing computational models of dopamine and noradrenaline dysfunction in attention deficit/hyperactivity disorder. Neuropsychopharmacology 32, 1583–1599 10.1038/sj.npp.1301278 17164816

[B54] Frank M. J. Seeberger L. C. O'Reilly R. C. (2004). By carrot or by stick: cognitive reinforcement learning in parkinsonism. Science 306, 1940–1943 10.1126/science.1102941 15528409

[B55] Gasper K. Clore G. L. (2002). Attending to the big picture: mood and global versus local processing of visual information. Psychol. Sci. 13, 34–40 10.1111/1467-9280.00406 11892776

[B56] Guitart-Masip M. Beierholm U. R. Dolan R. Duzel E. Dayan P. (2011a). Vigor in the face of fluctuating rates of reward: an experimental examination. J. Cogn. Neurosci. 23, 3933–3938 21736459 10.1162/jocn_a_00090

[B57] Guitart-Masip M. Fuentemilla L. Bach D. R. Huys Q. J. Dayan P. Dolan R. J. (2011b). Action dominates valence in anticipatory representations in the human striatum and dopaminergic midbrain. J. Neurosci. 31, 7867–7875 10.1523/JNEUROSCI.6376-10.2011 21613500 PMC3109549

[B58] Guitart-Masip M. Huys Q. J. Fuentemilla L. Dayan P. Duzel E. Dolan R. J. (2012). Go and no-go learning in reward and punishment: interactions between affect and effect. Neuroimage 62, 154–166 10.1016/j.neuroimage.2012.04.024 22548809 PMC3387384

[B59] Gur R. C. Ragland J. D. Reivich M. Greenberg J. H. Alavi A. Gur R. E. (2009). Regional differences in the coupling between resting cerebral blood flow and metabolism may indicate action preparedness as a default state. Cereb. cortex 19, 375–382 10.1093/cercor/bhn087 18534991 PMC2638785

[B60] Hampton A. N. Bossaerts P. O'Doherty J. P. (2006). The role of the ventromedial prefrontal cortex in abstract state-based inference during decision making in humans. J. Neurosci. 26, 8360–8367 10.1523/JNEUROSCI.1010-06.2006 16899731 PMC6673813

[B61] Handley I. M. Lassiter G. D. Nickell E. F. Herchenroeder L. M. (2004). Affect and automatic mood maintenance. J. Exp. Soc. Psychol. 40, 106–112 10.1016/S0022-1031(03)00086-6

[B62] Hanes D. P. Patterson W. F. Schall J. D. (1998). Role of frontal eye fields in countermanding saccades: visual, movement, and fixation activity. J. Neurophysiol. 79, 817–834 9463444 10.1152/jn.1998.79.2.817

[B63] Hare T. A. Tottenham N. Davidson M. C. Glover G. H. Casey B. J. (2005). Contributions of amygdala and striatal activity in emotion regulation. Biol. Psychiatry 57, 624–632 10.1016/j.biopsych.2004.12.038 15780849

[B64] Harlé K. M. Chang L. J. Van't Wout M. Sanfey A. G. (2012). The neural mechanisms of affect infusion in social economic decision-making: a mediating role of the anterior insula. Neuroimage 61, 32–40 10.1016/j.neuroimage.2012.02.027 22374480

[B65] Harlé K. M. Sanfey A. G. (2007). Incidental sadness biases social economic decisions in the Ultimatum Game. Emotion 7:876 10.1037/1528-3542.7.4.876 18039057

[B66] Harmon-Jones E. Allen J. J. B. (1998). Anger and frontal brain activity: EEG asymmetry consistent with approach motivation despite negative affective valence. J. Pers. Soc. Psychol. 74:1310 10.1037/0022-3514.74.5.1310 9599445

[B67] Henriques J. B. Davidson R. J. (2000). Decreased responsiveness to reward in depression. Cogn. Emot. 14, 711–724 10.1080/02699930050117684

[B68] Horga G. Maia T. V. Wang P. Wang Z. Marsh R. Peterson B. S. (2011). Adaptation to conflict via context-driven anticipatory signals in the dorsomedial prefrontal cortex. J. Neurosci. 31, 16208–16216 10.1523/JNEUROSCI.2783-11.2011 22072672 PMC3244974

[B69] Huys Q. J. Cools R. Gölzer M. Friedel E. Heinz A. Dolan R. J. (2011). Disentangling the roles of approach, activation and valence in instrumental and pavlovian responding. PLoS Comput. Biol. 7:e1002028 10.1371/journal.pcbi.1002028 21556131 PMC3080848

[B70] Ide J. S. Shenoy P. Yu A. J. Li C. S. (2013). Bayesian prediction and evaluation in the anterior cingulate cortex. J. Neurosci. 33, 2039–2047 10.1523/JNEUROSCI.2201-12.2013 23365241 PMC3711643

[B71] Johnson S. L. (2005). Mania and dysregulation in goal pursuit: a review. Clin. Psychol. Rev. 25, 241–262 10.1016/j.cpr.2004.11.002 15642648 PMC2847498

[B72] Joormann J. Gotlib I. H. (2006). Is this happiness I see? Biases in the identification of emotional facial expressions in depression and social phobia. J. Abnorm. Psychol. 115, 705 10.1037/0021-843X.115.4.705 17100528

[B73] Kahneman D. Tversky A. (1979). Prospect theory: an analysis of decision under risk. Econometrica 47, 263–291 10.2307/1914185

[B74] Kerns J. G. (2006). Anterior cingulate and prefrontal cortex activity in an FMRI study of trial-to-trial adjustments on the Simon task. Neuroimage 33, 399 10.1016/j.neuroimage.2006.06.012 16876434

[B75] Kerns J. G. Cohen J. D. Macdonald A. W. Cho R. Y. Stenger V. A. Carter C. S. (2004). Anterior cingulate conflict monitoring and adjustments in control. Science 303, 1023–1026 10.1126/science.1089910 14963333

[B76] Knutson B. Fong G. W. Adams C. M. Varner J. L. Hommer D. (2001). Dissociation of reward anticipation and outcome with event-related fMRI. Neuroreport 12, 3683–3687 10.1097/00001756-200112040-00016 11726774

[B77] Knutson B. Peterson R. (2005). Neurally reconstructing expected utility. Games Econ. Behav. 52, 305–315 10.1016/j.geb.2005.01.002

[B78] Kravitz A. V. Tye L. D. Kreitzer A. C. (2012). Distinct roles for direct and indirect pathway striatal neurons in reinforcement. Nat. Neurosci. 15, 816–818 10.1038/nn.3100 22544310 PMC3410042

[B79] Kross E. Egner T. Ochsner K. Hirsch J. Downey G. (2007). Neural dynamics of rejection sensitivity. J. Cogn. Neurosci. 19, 945–956 10.1162/jocn.2007.19.6.945 17536965

[B80] Kuhnen C. M. Knutson B. (2005). The neural basis of financial risk taking. Neuron 47, 763–770 10.1016/j.neuron.2005.08.008 16129404

[B81] Ladouceur C. D. Dahl R. E. Williamson D. E. Birmaher B. Axelson D. A. Ryan N. D. (2006). Processing emotional facial expressions influences performance on a Go/NoGo task in pediatric anxiety and depression. J. Child Psychol. Psychiatry 47, 1107–1115 10.1111/j.1469-7610.2006.01640.x 17076749

[B82] Lang P. J. Bradley M. M. Cuthbert B. N. (1997). Motivated attention: affect, activation, and action, in Attention and Orienting: Sensory and Motivational Processes, eds P. J. Lang, R. F. Simons, and M. T. Balaban (Hillsdale, NJ: Erlbaum), 97–135

[B83] Langenecker S. A. Kennedy S. E. Guidotti L. M. Briceno E. M. Own L. S. Hooven T. (2007). Frontal and limbic activation during inhibitory control predicts treatment response in major depressive disorder. Biol. Psychiatry 62, 1272–1280 10.1016/j.biopsych.2007.02.019 17585888 PMC2860742

[B84] Lennox B. Jacob R. Calder A. Lupson V. Bullmore E. (2004). Behavioural and neurocognitive responses to sad facial affect are attenuated in patients with mania. Psychol. Med. 34, 795–802 10.1017/S0033291704002557 15500300

[B85] Liddle E. B. Scerif G. Hollis C. P. Batty M. J. Groom M. J. Liotti M. (2009). Looking before you leap: a theory of motivated control of action. Cognition 112, 141–158 10.1016/j.cognition.2009.03.006 19409540 PMC2706947

[B86] Loewenstein G. Lerner J. S. (2003). The role of affect in decision making. Handb. Affect. Sci. 619, 642

[B87] Loftus E. F. Loftus G. R. Messo J. (1987). Some facts about “weapon focus.”. Law Hum. Behav. 11, 55 10.1007/BF01044839

[B88] Logan G. D. Cowan W. B. (1984). On the ability to inhibit thought and action: a theory of an act of control. Psychol. Rev. 91, 295 10.1037/0033-295X.91.3.295 24490789

[B89] Luu P. Collins P. Tucker D. M. (2000). Mood, personality, and self-monitoring: negative affect and emotionality in relation to frontal lobe mechanisms of error monitoring. J. Exp. Psychol. Gen. 129, 43 10.1037/0096-3445.129.1.43 10756486

[B90] Macleod C. M. Macdonald P. A. (2000). Interdimensional interference in the Stroop effect: uncovering the cognitive and neural anatomy of attention. Trends Cogn. Sci. 4, 383–391 10.1016/S1364-6613(00)01530-8 11025281

[B91] Maia T. V. (2010). Two-factor theory, the actor-critic model, and conditioned avoidance. Learn. Behav. 38, 50–67 10.3758/LB.38.1.50 20065349

[B92] Mathews A. Macleod C. (1994). Cognitive approaches to emotion and emotional disorders. Annu. Rev. Psychol. 45, 25–50 10.1146/annurev.ps.45.020194.000325 8135504

[B93] Miller A. Tomarken A. J. (2001). Task-dependent changes in frontal brain asymmetry: effects of incentive cues, outcome expectancies, and motor responses. Psychophysiology 38, 500–511 10.1111/1469-8986.3830500 11352139

[B94] Miyake A. Friedman N. P. Emerson M. J. Witzki A. H. Howerter A. Wager T. D. (2000). The unity and diversity of executive functions and their contributions to complex “frontal lobe” tasks: a latent variable analysis. Cogn. Psychol. 41, 49–100 10.1006/cogp.1999.0734 10945922

[B95] Montague P. R. Hyman S. E. Cohen J. D. (2004). Computational roles for dopamine in behavioural control. Nature 431, 760–767 10.1038/nature03015 15483596

[B96] Montague P. R. King-Casas B. Cohen J. D. (2006). Imaging valuation models in human choice. Annu. Rev. Neurosci. 29, 417–448 10.1146/annurev.neuro.29.051605.112903 16776592

[B97] Morris G. Nevet A. Arkadir D. Vaadia E. Bergman H. (2006). Midbrain dopamine neurons encode decisions for future action. Nat. Neurosci. 9, 1057–1063 10.1038/nn1743 16862149

[B98] Murphy F. Sahakian B. Rubinsztein J. Michael A. Rogers R. Robbins T. (1999). Emotional bias and inhibitory control processes in mania and depression. Psychol. Med. 29, 1307–1321 10.1017/S0033291799001233 10616937

[B99] Nee D. E. Wager T. D. Jonides J. (2007). Interference resolution: insights from a meta-analysis of neuroimaging tasks. Cogn. Affect. Behav. Neurosci. 7, 1–17 10.3758/CABN.7.1.1 17598730

[B100] Niv Y. Daw N. D. Joel D. Dayan P. (2007). Tonic dopamine: opportunity costs and the control of response vigor. Psychopharmacology 191, 507–520 10.1007/s00213-006-0502-4 17031711

[B101] Norton P. J. Asmundson G. J. (2004). Amending the fear-avoidance model of chronci pain: what is the role of physiological arousal? Behav. Ther. 34, 17–30 10.1016/S0005-7894(03)80019-9

[B102] O'Doherty J. P. (2004). Reward representations and reward-related learning in the human brain: insights from neuroimaging. Curr. Opin. Neurobiol. 14, 769–776 10.1016/j.conb.2004.10.016 15582382

[B103] Olvet D. M. Hajcak G. (2008). The error-related negativity (ERN) and psychopathology: toward an endophenotype. Clin. Psychol. Rev. 28, 1343–1354 10.1016/j.cpr.2008.07.003 18694617 PMC2615243

[B104] Overtoom C. Verbaten M. Kemner C. Kenemans J. Engeland H. V. Buitelaar J. (2003). Effects of methylphenidate, desipramine, and L-dopa on attention and inhibition in children with attention deficit hyperactivity disorder. Behav. Brain Res. 145, 7–15 10.1016/S0166-4328(03)00097-4 14529800

[B105] Pallak M. S. Pittman T. S. Heller J. F. Munson P. (1975). The effect of arousal on Stroop color-word task performance. Bull. Psychon. Soc. 6, 248–250

[B106] Paré M. Hanes D. P. (2003). Controlled movement processing: superior colliculus activity associated with countermanded saccades. J. Neurosci. 23, 6480–6489 12878689 10.1523/JNEUROSCI.23-16-06480.2003 PMC6740637

[B107] Paulus M. P. Feinstein J. S. Leland D. Simmons A. N. (2005). Superior temporal gyrus and insula provide response and outcome-dependent information during assessment and action selection in a decision-making situation. Neuroimage 25, 607–615 10.1016/j.neuroimage.2004.12.055 15784440

[B108] Paulus M. P. Feinstein J. S. Simmons A. Stein M. B. (2004). Anterior cingulate activation in high trait anxious subjects is related to altered error processing during decision making. Biol. Psychiatry 55, 1179–1187 10.1016/j.biopsych.2004.02.023 15184037

[B109] Paulus M. P. Stein M. B. (2006). An insular view of anxiety. Biol. Psychiatry 60, 383–387 10.1016/j.biopsych.2006.03.042 16780813

[B110] Pessoa L. (2009). How do emotion and motivation direct executive control? Trends Cogn. Sci. 13, 160–166 10.1016/j.tics.2009.01.006 19285913 PMC2773442

[B111] Pessoa L. Padmala S. Kenzer A. Bauer A. (2012). Interactions between cognition and emotion during response inhibition. Emotion 12, 192 10.1037/a0024109 21787074 PMC3208031

[B112] Peterson B. S. Kane M. J. Alexander G. M. Lacadie C. Skudlarski P. Leung H. C. (2002). An event-related functional MRI study comparing interference effects in the Simon and Stroop tasks. Cogn. Brain Res. 13, 427–440 10.1016/S0926-6410(02)00054-X 11919006

[B113] Phillips P. E. M. Walton M. E. Jhou T. C. (2007). Calculating utility: preclinical evidence for cost–benefit analysis by mesolimbic dopamine. Psychopharmacology 191, 483–495 10.1007/s00213-006-0626-6 17119929

[B114] Pizzagalli D. A. Sherwood R. J. Henriques J. B. Davidson R. J. (2005). Frontal brain asymmetry and reward responsiveness a source-localization study. Psychol. Sci. 16, 805–813 10.1111/j.1467-9280.2005.01618.x 16181444

[B115] Price R. B. Mohlman J. (2007). Inhibitory control and symptom severity in late life generalized anxiety disorder. Behav. Res. Ther. 45, 2628–2639 10.1016/j.brat.2007.06.007 17662240

[B116] Ramos B. P. Arnsten A. F. (2007). Adrenergic pharmacology and cognition: focus on the prefrontal cortex. Pharmacol. Ther. 113, 523–536 10.1016/j.pharmthera.2006.11.006 17303246 PMC2151919

[B117] Robbins T. Arnsten A. (2009). The neuropsychopharmacology of fronto-executive function: monoaminergic modulation. Annu. Rev. Neurosci. 32, 267 10.1146/annurev.neuro.051508.135535 19555290 PMC2863127

[B118] Robinson E. S. Eagle D. M. Mar A. C. Bari A. Banerjee G. Jiang X. (2007). Similar effects of the selective noradrenaline reuptake inhibitor atomoxetine on three distinct forms of impulsivity in the rat. Neuropsychopharmacology 33, 1028–1037 10.1038/sj.npp.1301487 17637611

[B119] Salamone J. D. Correa M. Farrar A. Mingote S. M. (2007). Effort-related functions of nucleus accumbens dopamine and associated forebrain circuits. Psychopharmacology 191, 461–482 10.1007/s00213-006-0668-9 17225164

[B120] Samanez-Larkin J. R. Hollon N. G. Cartensen L. L. Knutson B. (2008). Individual differences in insular Ssensitivity during loss anticipation predict avoidance learning. Psyhol. Sci. 19, 320–323 10.1111/j.1467-9280.2008.02087.x 18399882 PMC2365707

[B121] Samson R. Frank M. Fellous J.-M. (2010). Computational models of reinforcement learning: the role of dopamine as a reward signal. Cogn. Neurodyn. 4, 91–105 10.1007/s11571-010-9109-x 21629583 PMC2866366

[B122] Sara S. Segal M. (1991). Plasticity of sensory responses of locus coeruleus neurons in the behaving rat: implications for cognition. Prog. Brain Res. 88, 571–585 10.1016/S0079-6123(08)63835-2 1813935

[B123] Schachter S. Singer J. (1962). Cognitive, social, and physiological determinants of emotional state. Psychol. Rev. 69, 379 14497895 10.1037/h0046234

[B124] Schultz W. Dayan P. Montague P. R. (1997). A neural substrate of prediction and reward. Science 275, 1593–1599 10.1126/science.275.5306.1593 9054347

[B125] Schulz K. P. Fan J. Magidina O. Marks D. J. Hahn B. Halperin J. M. (2007). Does the emotional go/no-go task really measure behavioral inhibition? Convergence with measures on a non-emotional analog. Arch. Clin. Neuropsychol. 22, 151–160 10.1016/j.acn.2006.12.001 17207962 PMC2562664

[B126] Schutter D. J. Hofman D. Van Honk J. (2008). Fearful faces selectively increase corticospinal motor tract excitability: a transcranial magnetic stimulation study. Psychophysiology 45, 345–348 10.1111/j.1469-8986.2007.00635.x 18221448

[B127] Schwabe L. Wolf O. T. (2009). Stress prompts habit behavior in humans. J. Neurosci. 29, 7191–7198 10.1523/JNEUROSCI.0979-09.2009 19494141 PMC6666491

[B128] Schwarz N. Clore G. L. (1983). Mood, misattribution, and judgments of well-being: Informative and directive functions of affective states. J. Pers. Soc. Psychol. 45, 513 10.1037/0022-3514.45.3.513

[B129] Shadmehr R. (2010). Control of movements and temporal discounting of reward. Curr. Opin. Neurobiol. 20, 726–730 10.1016/j.conb.2010.08.017 20833031

[B130] Shenoy P. Jahfari S. Yu A. (2012). Differential efficiency of conflict resolution underlies individual differences in inhibitory capacity in the stop simon task, in Poster Presented at the Society for Neuroscience, (New Orleans, LA).

[B131] Shenoy P. Yu A. (2012). Strategic impatience in Go/NoGo versus forced-choice decision-making. Adv. Neural. Inf. Process Syst. 25, 2132–2140

[B132] Shenoy P. Yu A. J. (2011). Rational decision-making in inhibitory control. Front. Hum. Neurosci. 5:48 10.3389/fnhum.2011.00048 21647306 PMC3103997

[B133] Sinclair R. C. Hoffman C. Mark M. M. Martin L. L. Pickering T. L. (1994). Construct accessibility and the misattribution of arousal: schachter and Singer revisited. Psychol. Sci. 5, 15–19 10.1111/j.1467-9280.1994.tb00607.x

[B134] Somerville L. H. Heatherton T. F. Kelley W. M. (2006). Anterior cingulate cortex responds differentially to expectancy violation and social rejection. Nat. Neurosci. 9, 1007–1008 10.1038/nn1728 16819523

[B135] Storbeck J. Clore G. L. (2008). Affective arousal as information: how affective arousal influences judgments, learning, and memory. Soc. Pers. Psychol. Compass 2, 1824–1843 10.1111/j.1751-9004.2008.00138.x 25067943 PMC4110743

[B136] Stuphorn V. Brown J. W. Schall J. D. (2010). Role of supplementary eye field in saccade initiation: executive, not direct, control. J. Neurophysiol. 103, 801–816 10.1152/jn.00221.2009 19939963 PMC2822692

[B137] Swick D. Ashley V. Turken U. (2011). Are the neural correlates of stopping and not going identical? Quantitative meta-analysis of two response inhibition tasks. Neuroimage 56, 1655–1665 10.1016/j.neuroimage.2011.02.070 21376819

[B138] Tang N. K. Salkovskis P. M. Hodges A. Wright K. J. Hanna M. Hester J. (2008). Effects of mood on pain responses and pain tolerance: an experimental study in chronic back pain patients. Pain 138, 392–401 10.1016/j.pain.2008.01.018 18325674

[B139] Tellegen A. Watson D. Clark L. A. (1999). On the dimensional and hierarchical structure of affect. Psychol. Sci. 10, 297–303 10.1111/1467-9280.00157

[B140] Trevisani D. P. Johnson S. L. Carver C. S. (2008). Positive mood induction and facial affect recognition among students at risk for mania. Cogn. Ther. Res. 32, 639–650 10.1007/s10608-007-9140-3 20126422 PMC2814431

[B141] van Steenbergen H. Band G. P. Hommel B. (2009). Reward counteracts conflict adaptation evidence for a role of affect in executive control. Psychol. Sci. 20, 1473–1477 10.1111/j.1467-9280.2009.02470.x 19906127

[B142] van Steenbergen H. Band G. P. H. Hommel B. (2010). In the mood for adaptation how affect regulates conflict-driven control. Psychol. Sci. 21, 1629–1634 10.1177/0956797610385951 20943936

[B143] Verbruggen F. De Houwer J. (2007). Do emotional stimuli interfere with response inhibition? Evidence from the stop signal paradigm. Cogn. Emot. 21, 391–403 10.1080/02699930600625081

[B144] Weinberg A. Olvet D. M. Hajcak G. (2010). Increased error-related brain activity in generalized anxiety disorder. Biol. Psychol. 85, 472–480 10.1016/j.biopsycho.2010.09.011 20883743

[B145] Winkielman P. Knutson B. Paulus M. Trujillo J. L. (2007). Affective influence on judgments and decisions: moving towards core mechanisms. Rev. Gen. Psychol. 11, 179 10.1037/1089-2680.11.2.179

[B146] Wiswede D. Münte T. F. Rüsseler J. (2009). Negative affect induced by derogatory verbal feedback modulates the neural signature of error detection. Soc. Cogn. Affect. Neurosci. 4, 227–237 10.1093/scan/nsp015 19454619 PMC2728634

[B147] Yacubian J. Sommer T. Schroeder K. Gläscher J. Braus D. F. Büchel C. (2007). Subregions of the ventral striatum show preferential coding of reward magnitude and probability. Neuroimage 38, 557–563 10.1016/j.neuroimage.2007.08.007 17889562

[B148] Yu A. J. Dayan P. (2005). Uncertainty, neuromodulation, and attention. Neuron 46, 681–692 10.1016/j.neuron.2005.04.026 15944135

[B149] Yu A. J. Dayan P. Cohen J. D. (2009). Dynamics of attentional selection under conflict: toward a rational Bayesian account. J. Exp. Psychol. Hum. Percept. Perform. 35, 700 10.1037/a0013553 19485686 PMC3432507

[B150] Zhao H. Chen A. C. (2009). Both happy and sad melodies modulate tonic human heat pain. J. Pain 10, 953–960 10.1016/j.jpain.2009.03.006 19595640

